# Loss-of-function mutations in *UDP-Glucose 6-Dehydrogenase* cause recessive developmental epileptic encephalopathy

**DOI:** 10.1038/s41467-020-14360-7

**Published:** 2020-01-30

**Authors:** Holger Hengel, Célia Bosso-Lefèvre, George Grady, Emmanuelle Szenker-Ravi, Hankun Li, Sarah Pierce, Élise Lebigot, Thong-Teck Tan, Michelle Y. Eio, Gunaseelan Narayanan, Kagistia Hana Utami, Monica Yau, Nader Handal, Werner Deigendesch, Reinhard Keimer, Hiyam M. Marzouqa, Meral Gunay-Aygun, Michael J. Muriello, Helene Verhelst, Sarah Weckhuysen, Sonal Mahida, Sakkubai Naidu, Terrence G. Thomas, Jiin Ying Lim, Ee Shien Tan, Damien Haye, Michèl A. A. P. Willemsen, Renske Oegema, Wendy G. Mitchell, Tyler Mark Pierson, Marisa V. Andrews, Marcia C. Willing, Lance H. Rodan, Tahsin Stefan Barakat, Marjon van Slegtenhorst, Ralitza H. Gavrilova, Diego Martinelli, Tal Gilboa, Abdullah M. Tamim, Mais O. Hashem, Moeenaldeen D. AlSayed, Maha M. Abdulrahim, Mohammed Al-Owain, Ali Awaji, Adel A. H. Mahmoud, Eissa A. Faqeih, Ali Al Asmari, Sulwan M. Algain, Lamyaa A. Jad, Hesham M. Aldhalaan, Ingo Helbig, David A. Koolen, Angelika Riess, Ingeborg Kraegeloh-Mann, Peter Bauer, Suleyman Gulsuner, Hannah Stamberger, Alvin Yu Jin Ng, Sha Tang, Sumanty Tohari, Boris Keren, Laura E. Schultz-Rogers, Eric W. Klee, Sabina Barresi, Marco Tartaglia, Hagar Mor-Shaked, Sateesh Maddirevula, Amber Begtrup, Aida Telegrafi, Rolph Pfundt, Rebecca Schüle, Brian Ciruna, Carine Bonnard, Mahmoud A. Pouladi, James C. Stewart, Adam Claridge-Chang, Dirk J. Lefeber, Fowzan S. Alkuraya, Ajay S. Mathuru, Byrappa Venkatesh, Joseph J. Barycki, Melanie A. Simpson, Saumya S. Jamuar, Ludger Schöls, Bruno Reversade

**Affiliations:** 1 Department of Neurology and Hertie-Institute for Clinical Brain Research, University of Tübingen, Tübingen, Germany; 2 German Center of Neurodegenerative Diseases (DZNE), Tübingen, Germany; 3 Institute of Medical Biology, A*STAR, Biopolis, Singapore, 138648 Singapore; 4 National University of Singapore, Department of Paediatrics, Yong Loo Lin School of Medicine, Biopolis, Singapore, Singapore; 5 Department of Molecular and Structural Biochemistry North Carolina State University, Raleigh, NC 27607 USA; 6 Yale-NUS College, 12 College Avenue West, Biopolis, Singapore, Singapore; 7 Division of Medical Genetics, Department of Medicine, University of Washington, Seattle, WA USA; 8 Service De Biochimie, Hopital Bicêtre, Assistance publique-Hôpitaux de Paris, 78 avenue du general leclerc, Le Kremlin Bicêtre, France; 9 Institute of Medical Biology, Singapore Stem Cell Bank, A∗STAR, Biopolis, Singapore, 138648 Singapore; 10 Translational Laboratory in Genetic Medicine, Agency for Science, Technology, and Research, Singapore (A*STAR), 8A Biomedical Grove, Immunos, Level 5, Singapore, 138648 Singapore; 11 Program in Developmental & Stem Cell Biology, The Hospital for Sick Children, Department of Molecular Genetics, The University of Toronto, Toronto, ON Canada; 12 Caritas Baby Hospital Bethlehem, Bethlehem, State of Palestine; 13 Ped Neurology, Staufer Hospital, Wetzgauer Straße 85, Schwäbisch-Gmünd, Germany; 14 McKusick-Nathans Institute of Genetic Medicine, Johns Hopkins University School of Medicine, Baltimore, MD 21205 USA; 15 Department of Paediatric Neurology, Ghent University Hospital, Ghent, Belgium; 16 Center for Molecular Neurology, VIB, Antwerp, Belgium; 17 Laboratory of Neurogenetics, Institute Born-Bunge, University of Antwerp, Antwerp, Belgium; 18 Department of Neurology, University Hospital Antwerp, Antwerp, Belgium; 19 Division of Neurology and Neurogenetics, Kennedy Krieger Institute, Baltimore, MD USA; 20 Neurology Service, Department of Paediatrics, KK Women’s and Children’s Hospital, Singapore, Singapore; 21 Genetics Service, Department of Paediatrics, KK Women’s and Children’s Hospital, Singapore, Singapore; 22 Paediatric Academic Clinical Programme, Duke-NUS Medical School, Singapore, Singapore; 23 SingHealth Duke-NUS Genomic Medicine Centre, Singapore, Singapore; 24 Service de Génétique Médicale, CHU De Nice Hôpital de l’Archet 2, 151 route Saint Antoine de la Ginestière, CS 23079 062002, Nice, Cedex 3 France; 25 Department of Pediatric Neurology, Radboud University Medical Center, Nijmegen, The Netherlands; 26 Department of Genetics, University Medical Center Utrecht, Utrecht, The Netherlands; 27 Neurology Division, Childrens Hospital Los Angeles & Department of Neurology, Keck School of Medicine of University of Southern California, Los Angeles, CA 90033 USA; 28 Department of Pediatrics, Department of Neurology, & the Board of Governors Regenerative Medicine Institute, Cedars-Sinai Medical Center, Los Angeles, CA USA; 29 Division of Genetics and Genomic Medicine, Department of Pediatrics, Washington University School of Medicine, St. Louis, MO USA; 30 Division of Genetics and Genomics and Department of Neurology, Boston Children’s Hospital, Boston, MA USA; 31 Department of Clinical Genetics, Erasmus MC, University Medical Center, Wytemaweg 80, 3015 CN Rotterdam, The Netherlands; 32 Department of Clinical Genomics, Mayo Clinic, 200 First Street SW, Rochester, MN USA; 33 Genetics and Rare Diseases Research Division, Ospedale Pediatrico Bambino Gesù, IRCCS, viale San Paolo 15, 00146 Rome, Italy; 34 Child Neurology Unit, Hadassah-Hebrew University Medical Center, 9112001 Jerusalem, Israel; 35 Pediatric Neurology, King Faisal Specialist Hospital and Research Center, Riyadh, Saudi Arabia; 36 Department of Genetics, King Faisal Specialist Hospital and Research Center, Riyadh, Saudi Arabia; 37 Department of Medical Genetics, King Faisal Specialist Hospital and Research Center, Riyadh, Saudi Arabia; 38 Department of Pediatrics, King Fahad Central Hospital in Jizan, Abu Arish, Saudi Arabia; 39 Pediatric Neurology Department, National Neuroscience Institute, King Fahad Medical City, Riyadh, Saudi Arabia; 40 Section of Medical Genetics, Children’s Hospital, King Fahad Medical City, Riyadh, Saudi Arabia; 41 General Pediatrics and Adolescents, King Fahad Medical City, Riyadh, Saudi Arabia; 42 Neuroscience Department King Faisal Specialist Hospital and Research Center, Riyadh, Saudi Arabia; 43 Division of Neurology, The Children’s Hospital of Philadelphia, Philadelphia, PA USA; 44 Department of Human Genetics, Radboud University Medical Center, Nijmegen, The Netherlands; 45 Institute of Medical Genetics and Applied Genomics (Tübingen) and Centogene AG (Rostock), Rostock, Germany; 46 Department of Pediatric Neurology, University of Tübingen, Tübingen, Germany; 47 Institute of Molecular and Cell Biology, A*STAR, Biopolis, Singapore, 138673 Singapore; 48 Division of Clinical Genomics, Ambry Genetics, Aliso Viejo, CA USA; 49 APHP, GH Pitié Salpêtrière, Department of Genetics, Unit of Development Genomics, Paris, France; 50 Department of Genetic and Metabolic Diseases, Hadassah-Hebrew University Medical Center, 9112001 Jerusalem, Israel; 51 GeneDx, 207 Perry Parkway, Gaithersburg, MD 20877 USA; 52 Department of Physiology, National University of Singapore, Singapore, 117597 Singapore; 53 Department of Medicine, National University of Singapore, Singapore, 117597 Singapore; 54 Program in Neuroscience and Behavioral Disorders, Duke-NUS Medical School, Singapore, Singapore; 55 Department of Neurology, Donders Center for Brain, Cognition, and Behavior, Nijmegen, The Netherlands; 56 Department of Laboratory Medicine, Translational Metabolic Laboratory, Nijmegen, The Netherlands; 57 SingHealth Duke-NUS Institute of Precision Medicine, Singapore, Singapore; 58 Medical Genetics Department, Koç University School of Medicine, 34010 Istanbul, Turkey; 59 Reproductive Biology Laboratory, Obstetrics and Gynaecology, Academic Medical Center (AMC), Meibergdreef 9, 1105 AZ Amsterdam-Zuidoost, The Netherlands

**Keywords:** Glycobiology, Clinical genetics, Neuronal development, Encephalopathy

## Abstract

Developmental epileptic encephalopathies are devastating disorders characterized by intractable epileptic seizures and developmental delay. Here, we report an allelic series of germline recessive mutations in *UGDH* in 36 cases from 25 families presenting with epileptic encephalopathy with developmental delay and hypotonia. *UGDH* encodes an oxidoreductase that converts UDP-glucose to UDP-glucuronic acid, a key component of specific proteoglycans and glycolipids. Consistent with being loss-of-function alleles, we show using patients’ primary fibroblasts and biochemical assays, that these mutations either impair UGDH stability, oligomerization, or enzymatic activity. In vitro, patient-derived cerebral organoids are smaller with a reduced number of proliferating neuronal progenitors while mutant *ugdh* zebrafish do not phenocopy the human disease. Our study defines UGDH as a key player for the production of extracellular matrix components that are essential for human brain development. Based on the incidence of variants observed, *UGDH* mutations are likely to be a frequent cause of recessive epileptic encephalopathy.

## Abstract (web summary)

UDP-glucuronic acid is a component of the extracellular matrix. Here, the authors report biallelic variants in the gene encoding UDP-Glucose 6-Dehydrogenase (UGDH) in individuals affected by developmental epileptic encephalopathies that impair UGDH stability, oligomerization, or enzymatic activity in vitro.

## Introduction

Developmental epileptic encephalopathies are a clinically and genetically heterogeneous group of devastating disorders characterized by severe epileptic seizures that are accompanied by developmental delay or regression^1^. In several cases, a genetic etiology has been identified^2^. Germline mutations in these genes lead to different pathophysiological defects^2^, including ion channel dysfunction, synaptic impairment, transporter defects and metabolic abnormalities, such as deficiencies in glycosylation pathways^3–5^. However, the genetic cause of many epileptic encephalopathies remains unknown.

Defects of glycosylation are causing more than 100 rare human genetic disorders, most of these affecting the central and/or peripheral nervous systems. Patients typically show developmental delay or intellectual disability, seizures, neuropathy, and metabolic abnormalities in multiple organ systems^3^. Adding the correct sugar chains (glycans) to proteins and lipids significantly impacts their function. *UGDH* (MIM603370) codes for an enzyme that converts UDP-glucose (UDP-Glc) to UDP-glucuronic acid (UDP-GlcA) through the concomitant reduction of NAD^+^ into NADH^6,7^. UDP-GlcA is not only needed for detoxification via glucuronidation, but is also an obligate precursor for the synthesis of glycosaminoglycans (GAGs), and therefore an important component of proteoglycans of the extracellular matrix.

In this study, we establish *UGDH* as a gene responsible for autosomal recessive developmental epileptic encephalopathy in humans. We catalog a series of 30 patients from 25 families with biallelic germline *UGDH* variants. Using patients’ primary fibroblasts and biochemical assays, we demonstrate that these are loss-of-function alleles. While mutant *ugdh* zebrafish did not phenocopy the disease, we bring evidence that patient-derived cerebral organoids, which were smaller due to a reduced number of proliferating neuronal progenitors, can serve as an alternative disease-in-a-dish model for in vitro functional studies.

## Results

### Biallelic mutations in *UGDH* cause developmental epileptic encephalopathy

**Fig. 1:**
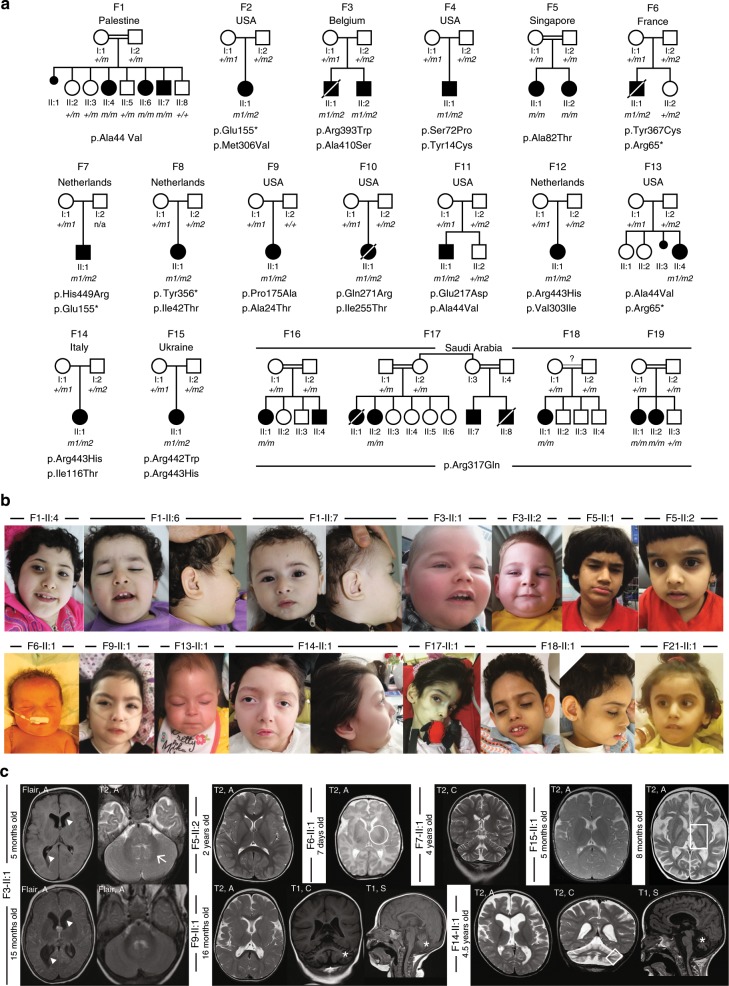
Clinical and genetic findings in 21 affected individuals diagnosed with Jamuar Syndrome consisting of developmental epileptic encephalopathy. **a** Pedigrees of 19 families segregating autosomal recessive developmental epileptic encephalopathy. Countries of origin are specified above each pedigree. Filled black symbols, affected individuals. Crossed symbols, deceased individual. Mutations in UGDH protein are presented below pedigrees. Homozygous mutations are presented in bold (*m* in the pedigrees). Compound heterozygous mutations are presented according to the parental origin of the mutation with a maternal origin in the first row (*m1* in the pedigrees), and a paternal, de novo or unknown origin in the second row (*m2* in the pedigrees). Healthy siblings that could be sequenced are heterozygous [F6-II:2 (p.Arg65*), F11-II:2 (p.Ala44Val), and F18-II:3 (p.Arg317Gln)]. **b** Facial photographs of 14 affected individuals with mild craniofacial dysmorphisms, including short and flattened philtrum, protruding earlobes, ptosis blepharophimosis, and epicanthic folds. **c** Spectrum of MRI findings in exemplary patients showing no evidence for maldevelopment but displaying variable abnormalities ranging from abnormal myelination and/or cerebral or cerebellar atrophy, to normal findings. Patient F5-II:2 presented with a normal MRI, including normal myelination at 2 years of age. In contrast, MRI of patient F3-II:1 revealed some myelination of cerebellar peduncles at 5 months (arrow) and no progress of myelination on follow-up at 15 months, indicative of hypomyelination. In addition, repeated MRI revealed enlarged posterior ventricles over time (arrow heads). MRI of patient F6-II:1 at 7 days of age also proved normal, the circle indicates onset of myelination in the Posterior Limb of the Internal Capsule (PLIC) according to age. Patient F7-II:1 showed mild cerebellar atrophy at 4 years of age. Patient F9-II:1 showed slightly delayed myelination on axial T2 and cerebellar atrophy on coronal and sagittal T1 images (stars). Patient F14-II:1 showed a diffuse cerebral atrophy, ventriculomegaly, thin corpus callosum, vermian, and lobar cerebellar atrophy, with normal brainstem, hyperintensity of cerebellar cortex in T2-weighted images (white square). Patient F15-II:1 presented with normal MRI at 5 months, but with severe diffuse atrophy, bilateral symmetrical hyperintensities of thalami and globus pallidus (white square) at 8 months old. In all pictures, MRI pulse sequences (T1, T2, and Flair) and image orientation (S: sagittal, A: axial and C: coronal) are indicated in the upper left corner.

To identify the genetic cause of a developmental epileptic encephalopathy in a consanguineous Palestinian family with three affected siblings (Fig. 1a, F1), we performed exome sequencing on two affected siblings. No mutations in genes known to be associated with neurological disorders (either recessive or dominant) were found. As the consanguineous background and the pedigree suggested autosomal recessive inheritance, we focused on homozygous or compound heterozygous variants shared by the affected siblings. A rare homozygous variant c.131C > T in *UDP-Glucose 6-Dehydrogenase* (*UGDH)*, which changes alanine into valine at position 44 of the UGDH protein, was the only segregating candidate variant. The *UGDH* p.A44V missense affects a highly conserved residue (Suppl. Fig. 1b and phyloP 100-way^8^ score 9.43), is extremely rare in public databases (not present in EVS6500^9^, MAF of 0.0017% in ExAC^10^) and is a good candidate according to in silico prediction scores (CADD score^11^ of 33) (Suppl. Table 1). We then (i) screened the GENESIS^12^ database for additional patients with recessive *UGDH* variants, (ii) contacted the EuroEPINOMICS RES Consortium, and (iii) searched with the help of GeneMatcher^13^ for additional families with germline *UGDH* mutations. We uncovered 27 additional patients from 24 families carrying either compound heterozygous or homozygous *UGDH* variants (Fig. 1a and Suppl. Fig. 1a). All variants were absent or had an extremely low frequency (<0.01%) in the public databases ExAC/gnomAD^10^ and EVS6500 (Suppl. Table 2). Nineteen of the 20 identified missense variants are in highly conserved residues (Suppl. Fig. 1b and phyloP 100-way between 3.81 and 9.43). The A44V variant, identified in the Palestinian index family, was also found in two additional families from Puerto Rico (F11) and from Spain (F13) indicative of independent but recurrent mutation in this residue. In ExAC the A44V variant is observed in African (MAF 0.0096%) and European (Non-Finish) populations (MAF 0.0015%), however, it is not present in the Greater Middle East Variome.

All 30 patients carrying biallelic mutations in *UGDH* presented with a common core phenotype consisting of marked developmental delay, epilepsy, mild dysmorphism, and motor disorder with axial hypotonia (Table 1 and Suppl. Data 1). Dysmorphic facial features such as short and flattened philtrum, outward protruding earlobes, ptosis, or blepharophimosis were mild but frequently present (Fig. 1b and Suppl. Data 1). Most patients have severe epilepsy ranging from neonatal onset developmental epileptic encephalopathy to infantile developmental epileptic encephalopathy (27 patients, 90%), of which 16 (53%) had infantile spasms (Table 1). Three patients have developmental encephalopathy, of which two had seizures in the setting of fever (F5-II:1 and F5-II:2). Only these two patients were seizure-free on sodium valproate. All other patients, except for one patient who seemed to benefit from ketogenic diet, did not respond to antiepileptic treatment. All patients had a severe motor disorder with axial hypotonia, while some patients presented with limb spasticity (43%), dystonia (17%), ataxia, chorea, and tremor, which were often present prior to onset of seizures. Twenty-four out of the 30 (80%) were noted to have swallowing difficulties and gastrostomy tubes were required for feeding in 12 infants. None but two patients (F5-II:1 and II:2) achieved sitting ability. A moderate to severe intellectual disability was observed in all patients. Three patients were deceased between 4 months and 6 years of age (Table 1 and Suppl. Data 1). Electroencephalography (EEG) was markedly abnormal with a burst suppression pattern in the neonatal period, hypsarrhythmia in affected children with infantile spasms, and focal and/or generalized spike-wave complexes in childhood (Suppl. Data 1). MRI revealed a spectrum of abnormalities with delayed myelination and enlarged ventricles probably due to cerebral and cerebellar atrophy in more severely affected patients without any signs of maldevelopment (Fig. 1c and Suppl. Data 1).

**Table 1:** Simplified clinical findings and course of disease in patients with *UGDH* mutations from families F1 to F10.

Family	Patient	Gender, age at last follow-up	Main phenotype	Age at seizure onset	Epilepsy, seizure types	Drug sensitivity	Motor development at last follow-up	Intellectual disability	Speech	Swallowing/ feeding difficulties	Hypotonia
F1	II:4	F, 13 yrs	IDEE	9 mths	Epileptic spasms	Resistant	Absence	Severe	Absence	Yes, open mouth	Yes
F1	II:6	M, 5 yrs	IDEE	15 mths	Epileptic spasms	Resistant	Absence	Severe	Absence	Yes, open mouth drooling	Yes
F1	II:7	M, 4 yrs	IDEE	6 mths	Epileptic spasms	Resistant	Absence	Severe	Absence	Yes, open mouth drooling	Yes
F2	II:1	F, 23 mths	IDEE	5 mths	Epileptic spasms reported back arching directyl after birth	Resistant	Absence	Severe	ND	ND	Yes
F3	II:1	M, 6 yrs^a^	IDEE	8 wks	Epilepsy with focal seizures, myoclonic jerks, epileptic spasms, status epilepticus	Resistant	Absence	Severe	Absence	Yes, g-tube	Yes
F3	II:2	M, 2 yrs	IDEE	4 mths	Epileptic spasms	Resistant	Absence	Severe	Absence	Yes, g-tube	Yes
F4	II:1	M, 5 yrs	IDEE	4 mths	Epileptic spasms	ND	Absence	Severe	Absence	Yes, g-tube	Yes
F5	II:1	F, 14 years	ID, MD	3 yrs	Seizures in the setting of fever	Seizure-free on sodium valproate	Sitting at 12 mths, walking at 3 yrs	Moderate	Slow acquisition, only single words at 14 yrs	Yes	Yes
F5	II:2	F, 6 yrs	ID, MD	3 yrs	Infrequent seizures in the setting of fever	Seizure-free on sodium valproate	Sitting unsupported at 23 mths, walking at 3 yrs	Moderate	First words at 18 mths, simple phrases at 6 yrs	Yes	Yes
F6	II:1	M, 4 mths^a^	NDEE	First day of life	Epileptic spasms	ND	ND	Severe	ND	Yes	Yes
F7	II:1	M, 7 yrs	IDEE	14 mths	Epileptic spasms, gelastic seizures, and other complex partial seizures	Resistant	Sitting with support at 14 mths	Severe	Absence	Yes, drooling, g-tube	Yes
F8	II:1	F, 25 mths	IDEE	12 mths	Epileptic spasms, stimulus-sensitive startles	Good response to ketogenic diet	Absence	Severe	Absence	Yes, g-tube	Yes
F9	II:1	F, 4 yrs	IDEE	8 wks	Epileptic spasms, myoclonic seizures, tonic seizures, clonic seizures	ND	Absence	Severe	Absence	Yes	Yes
F10	II:1	F, 5 mths^a^	IDEE, multiple congenital anomalies^b^	4 mths	Epileptic spasms	Resistant	Absence	Severe	Absence	Yes, g-tube	Yes
F11	II:1	M, 16 mths	IDEE	3 mths	Epileptic spasm, myoclonic seizure, tonic seizure, atonic seizure, clonic seizures	ND	Absence	Severe	Absence	Yes, NJ fed	Yes
F12	IV:3	F, 8 mths	NDEE	First day of life	Postpartum jitteryness, myoclonic jerks, and epileptic spasms	ND	ND	Severe	ND	Yes, g-tube	Yes
F13	II:4	F, 13 mths	IDEE	2 mths	Clusters of epileptic spasms	Resistant	Absence	Severe	ND	Yes, g-tube	Yes
F14	II:1	F, 8 yrs	IDEE	4 mths	Segmental and synchronous myoclonus, epileptic spasms in flexion	Resistant	Absence	Severe	Absence	Yes, g-tube	Yes
F15	II:1	F, 8 mths	IDEE	4 mths	Epileptic spasms	Resistant	Absence	Severe	Absence	Yes	Yes
F16	II:1	F, 11 yrs	IDEE	12 mths	Daily generalized tonic and myoclonic seizures	Resistant	Absence	Severe	Absence	Yes, g-tube	Yes
F17	II:2	F, 5 yrs	ID, MD	None	No epilepsy	n/a	Absence	Severe	Absence	No	Yes
F18	II:1	F, 8 yrs	IDEE	20 mths	Daily generalized tonic clonic and later myoclonic seizures with eye fluttering	Resistant	Absence	Severe	Absence	No	Yes
F19	II:1	F, 5 yrs	IDEE	30 mths	Recurrent generalized tonic clonic convulsions	ND	Absence	Severe	Absence	No	Yes
F19	II:2	F, 3 yrs	IDEE	18 mths	Daily myoclonic seizures with eye fluttering	ND	Absence	Severe	Absence	ND	Yes
F20	II:2	M, 6 yrs	IDEE	3 yrs	Epileptic spasms,myoclonic seizure, and tonic seizure	ND	Absence	Severe	Absence	Yes with difficulty	Yes
F21	II:2	F, 4 yrs	IDEE	18 mths	Myoclonic seizure	ND	Absence	Severe	Absence	Yes with difficulty	Yes
F22	II:5	F, 9 yrs	IDEE	20 mths	Epileptic spasms	Resistant	Absence	Severe	Absence	Yes, NJ fed	ND
F23	II:5	F, 7 yrs	IDEE	5 mths	Seizures in the setting of fever	ND	ND	Severe	Delay	ND	Mild
F24	II:1	M, 7 yrs	IDEE	6 mths	Myoclonic seizures, generalized tonic clonic seizures	Resistant	Absence	Severe	Absence	Yes, g-tube	Yes
F25	II:4	M, 8 yrs	IDEE	11 mths	Myoclonic seizures, generalized tonic clonic seizures	Resistant	Absence	Severe	Absence	Yes, g-tube	Yes

*M* male, *F* female, *IDEE* infantile developmental epileptic encephalopathy, *NDEE* neonatal onset developmental epileptic encephalopathy, *MD* motor disorder, *n/a* not applicable, *ND* non-determined, *ID* intellectual disability, *g-tube* gastrostomy tube, *NJ* nasojejunal, *wks* weeks, *mths* months, *yrs* years.

^a^Age at death.

^b^Multiple congenital anomalies in F16-II:1: prenatal polyhydramnios; multiple ocular anomalies (bilateral cataracts, multiple bilateral lens colobomas, bilateral microphthalmia, hypoplastic iris, iris and lenticular vascularization, bilateral anterior segment dysgenesis, posterior synechiae bilaterally secondary to neovascularization); megacystis; neurogenic bladder; moderate hiatal hernia; camptodactyly of 3rd and 4th fingers and overriding 2nd and 4th digits on the right hand; long tapered fingers; skeletal survey showed overlapping of the parietal bones and mild elongation of the 2nd through 5th fingers.

### *UGDH* mutations behave as hypomorphic alleles

**Fig. 2:**
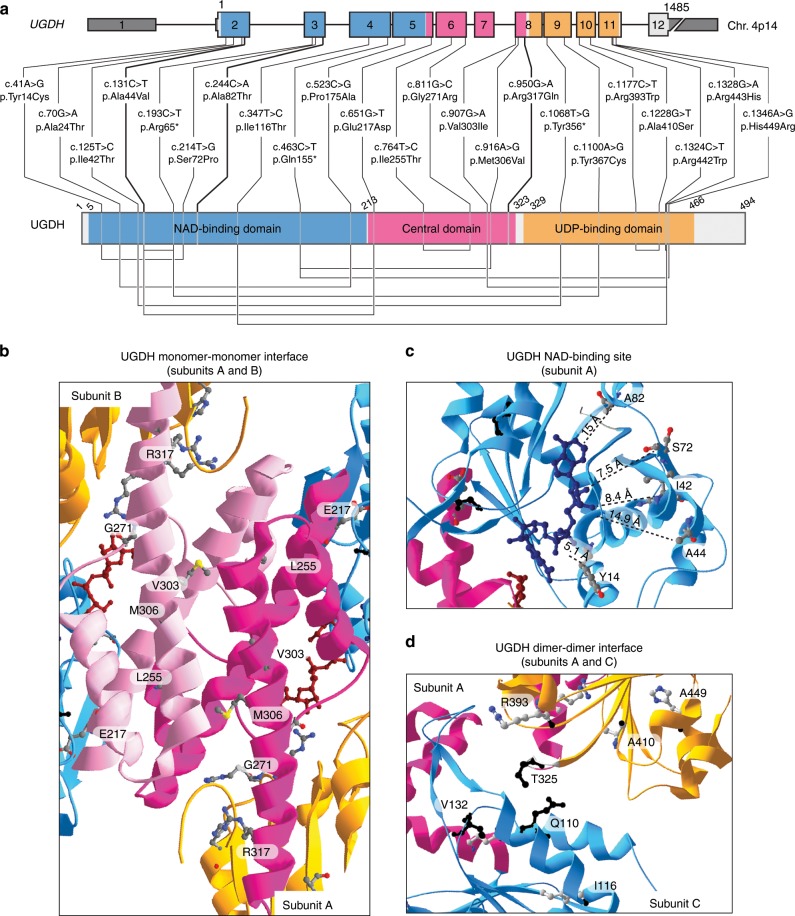
Mutations in UGDH enzyme possibly affect critical amino-acids. **a** *UGDH* genomic and protein domain structures. Type and positions of 22 germline *UGDH* mutations. 5′ and 3′ UTRs are shown in dark gray. NAD-binding (blue), central (pink), and UDP-binding (orange) domains are highlighted. Homozygous mutations are shown in bold. Compound heterozygous mutations that are in trans are linked by a line below the UGDH domain structure. **b**–**d** Three close-up views ribbon diagrams of the UGDH protein bound to UDP-Glc and NADH. **b** Interface between the central domains of subunits A and B. **c** NAD-binding site in NAD-binding domain of subunit A. Distances between NADH and mutated residues in patients are measured in Angström (Å). **d** Interface between the subunit A NAD-binding domain with the subunit C UDP-Glc-binding domain. In all the structures, residues carrying missense mutations in the patients are highlighted as 3D backbone. Residues Q110 and T325 known to interact together for dimer formation^15^; and residue V132, which is important for hexamerization^15^ are highlighted in black backbone. In all the structures, NAD-binding (blue), central (light/dark pink), and UDP-binding (orange) domains are shown. UDP-Glc (dark red) and NADH (midnight blue) are represented as colored carbon backbones. Adapted from PDB code 2Q3E^6^ using the Swiss-Pdb Viewer software^67^. For gels and graphs source data, please refer to the source data files 1 and 2.

The UGDH oxidoreductase consists of three distinct domains^14^: the NAD-binding (N-terminal) and UDP-binding (C-terminal) domains, and an internal domain that bridges the two termini together^14^. The UGDH enzyme assembles into a disc-shaped double layer composed of a trimer of dimers^6^ (Suppl. Fig. 2a, b). This hexameric structure is a prerequisite for proper UGDH enzymatic function^15^. The 23 germline mutations presented in this study are distributed throughout the *UGDH* gene and its encoded protein (Fig. 2a). One of the variants in Family 12 mutates the first nucleotide of exon 8 (c.907 G > A; p.Val303Ile, Fig. 2a, b), which is predicted to affect the splice donor site^16^. Three different nonsense mutations were found in a compound heterozygous state with a missense mutation (Fig. 2a and Suppl. Table 2). All identified missense mutations are anticipated to be destabilizing according to DUET^17^ (Suppl. Table 2, ΔΔG). The missense mutations in residues Y14, I42 and A44, which are close to the NAD-binding site (Fig. 2c and Suppl. Fig. 2c) are expected to impair NAD^+^ reduction. Alteration of residues in the central domain such as I255, G271, M306, and R317 are expected to affect homo-dimerization^18^ (Fig. 2b and Suppl. Fig. 2d). The I116 residue (located in the NAD-binding domain), as well as the R393 and A410 residues (UDP-Glc binding domain) sit at the dimer-dimer interface^19^ (Fig. 2d), suggesting that these variants may prevent UGDH from assembling into a functional hexameric enzyme.

**Fig. 3:**
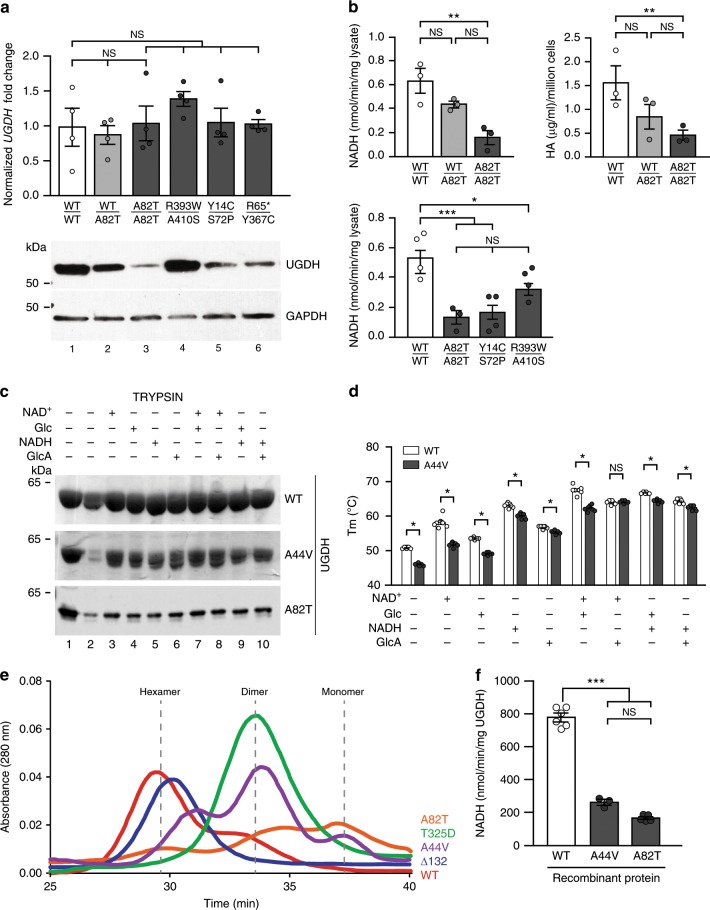
Biallelic UGDH mutations behave as hypomorphic alleles. **a** RT-qPCR (top), western blotting (bottom), and **b** enzymatic activity, assessed by measuring NADH production (left panel) and quantification of HA (right panel), for endogenous UGDH using patient-derived primary fibroblasts. **a**, **b** Control (WT/WT), unaffected mother F5-I:1 (WT/A82T) and 4 (in **a**) or 3 (in **b**) different patients’ fibroblasts (F5-II:1: A82T/A82T, F3-II:1: R393W/A410S, F4-II:1: Y14C/S72P, and F6-II:1: R65*/Y367C). **a** (top) Endogenous *UGDH* mRNA levels are normalized to *β-ACTIN* and *GAPDH*. Fold change relative to control (WT/WT) is plotted. **a** (bottom) Western blot analysis for endogenous UGDH protein using cellular extracts. GAPDH is used as a loading control. **b** (left) UGDH enzymatic activity measured as the conversion of NAD^+^ to NADH in whole-cell lysates. **b** (right) UGDH enzymatic activity measured as the HA production in conditioned media from primary fibroblast cultures. **c** Western blot analysis for UGDH sensitivity to limited proteolysis using purified WT and mutant (A44V and A82T) UGDH proteins in the absence or presence of its substrates and/or cofactors, as indicated. Results are representative of at least three experimental replicates. **d** Purified UGDH WT and A44V melting temperature (Tm) in the absence or presence of its substrates and/or cofactors, as indicated. Mean of three experiments ± S.D. is plotted for the *T*_m_ of each enzyme. **e** Representative traces at *λ* = 280 nm of purified WT and mutant UGDH proteins fractionated by size exclusion chromatography. WT, obligate dimer ∆132^15^, obligate hexamer T325D^15^, A44V and A82T UGDH are plotted in the graph. Dashed lines correspond to the known hexamer, dimer and monomer peak elution times. **f** Purified WT, A44V, and A82T UGDH enzymatic activity measured as the conversion of NAD + to NADH. Asterisks indicate *p*-values of *p* < 0.05(*), *p* < 0.01(**), and *p* < 0.001(***), NS: non-significant (*p* > 0.05) as determined by Student *t*-test. For gels and graphs source data, please refer to the source data files 1 and 2.

To better understand the effect of the mutations on UGDH, we then derived and biobanked primary dermal fibroblasts from patients F3-II:1 (R393W/A410S), F4-II:1 (Y14C/S72P), F5-II:1 (A82T/A82T) and F6-II:1 (R65*/Y367C), and a non-affected parent F5-I:1 (WT/A82T). Endogenous *UGDH* messenger RNA (mRNA) levels were not significantly different in patients’ primary cells as compared to control fibroblasts (Fig. 3a, top panels). In contrast, we observed significant changes in endogenous UGDH protein levels for three of the four alleles studied. Fibroblasts with compound heterozygous R393W/A410S mutations displayed comparable UGDH levels relative to wild-type (WT) cells, while patients’ cells with R65*/Y367C, Y14C/S72P, or homozygous A82T mutations showed dramatically reduced endogenous UGDH levels (Fig. 3a, bottom panels). In contrast to the three nonsense mutations and the missense mutation potentially affecting splicing, which are likely to cause nonsense-mediated decay of the endogenous *UGDH* transcript, the missense mutations are most likely impacting the stability of the enzyme and/or its oxidoreductase activity. Consistently, we observed a significant decrease in the UGDH-catalyzed reduction of NAD^+^ to NADH in patients’ primary fibroblasts (R393W/A410S, Y14C/S72P, or homozygous A82T mutations) while the non-affected parent’s cells heterozygous for the A82T mutation showed intermediate level of NAD^+^ reduction (Fig. 3b, left panels). Patient’s cells with the homozygous A82T mutation also exhibited a reduction in the synthesis of hyaluronic acid (HA), which requires UDP-glucuronate, a product of UGDH enzymatic activity (Fig. 3b, right panel). When produced in bacteria, mutant UGDH had altered stability, kinetic, and biochemical properties as compared to WT-UGDH. Compared to the wild-type enzyme, mutant A44V and A82T UGDH (mutations found at the homozygous state in the patients from Families 1 and 5, respectively) were more susceptible to partial proteolysis by trypsin (Fig. 3c). The stability of UGDH^A44V^ could be partially rescued upon incubation with substrate, product, or cofactor, while the UGDH^A82T^ remained strongly sensitive to proteolysis regardless of the presence of any cofactor or substrate (Fig. 3c). A thermal stability study showed that the melting temperature of UGDH^A44V^ was significantly reduced relative to WT and could only partially be rescued upon addition of substrate, product, reduced or oxidized cofactor, or any combination thereof (Fig. 3d). Notably, UGDH^A82T^ was so intrinsically unstable that a melting temperature was unable to be ascertained. By gel filtration chromatography, we investigated the effect of the A44V and A82T mutations on UGDH oligomerization. When compared to UGDH^WT^, UGDH^∆132^ (an obligate hexamer^15^), and UGDH^T325D^ (an obligate dimer^15^), we observed that UGDH^A44V^ and UGDH^A82T^ proteins were mainly eluted as dimer and monomer species, respectively, with virtually no stable hexameric population (Fig. 3e). This suggests that the A44V and A82T mutations may affect UGDH function by altering its capacity to form active hexamers. Finally, using equal amounts of recombinant enzyme, we determined that UGDH^A44V^ and UGDH^A82T^ were respectively 75 and 50% less efficient at reducing NAD^+^ to NADH as compared to UGDH^WT^ (Fig. 3f). Similarly, comparison of the steady state Michaelis–Menten kinetic constants (summarized in Table 2) showed that UGDH^A44V^ *V*_max_ was only ~ 50% of the value of UGDH^WT^ for both cofactor and substrate. In contrast, *K*_m_ was not significantly different from UGDH^WT^ for either cofactor or substrate, revealing that the mutation results in a reduced ability of the enzyme to catalyze the reaction, while still being able to associate with NAD^+^ and UDP-Glc. Taken together, our biochemical findings indicate that these missense mutations mainly impact the enzymatic function of UGDH by altering its quaternary structure and/or directly impairing its oxidoreductive activity.

**Table 2:** Summary of WT and mutant UGDH kinetic constants.

	*K*_m_ (µM)	*V*_max_ (nmol/min/mg)
UDP-glucose		
WT	28.6 ± 6.8	235.6 ± 12.5
A44V	15.9 ± 2.3*	118.3 ± 3.3***
A82T	ND	ND
NAD+		
WT	401 ± 75	219.2 ± 11.2
A44V	316 ± 54 NS	109.4 ± 4.5***
A82T	ND	ND

Steady state rate constants for UGDH^WT^ and UGDH^A44V^ were determined by varying UDP-glucose or NAD^+^ independently and fitting to the Michaelis–Menten equation. UGDH^A82T^ steady state constants could not be determined. Values indicate mean ± SD of triplicate assays. Asterisks indicate *p*-values of *p* < 0.05(*) and *p* < 0.001(***), NS: non-significant (*p* > 0.05) as determined by Student *t-*test. ND: not determined.

### Patient-derived cerebral organoids partially phenocopy human disease

Our attempts to model this developmental epileptic encephalopathy using the existing zebrafish hypomorphic loss-of-function *ugdh* (c.992 T > A; p.I331D) allele known as *jekyll m151*^20–22^ were unsuccessful (Suppl. Fig. 3). The behavioral activity of homozygous *jekyll* mutant larvae were recorded in presence or absence of the seizure-inducing drug pentylenetetrazol (PTZ)^23^. By quantitative PCR (qPCR), *c-fos* expression, which marks neural activity^23,24^, similarly increased in a dose-dependent manner upon PTZ treatment in all larvae regardless of genotypes (Suppl. Fig. 3a–d). Homozygous mutant larvae did not show signs of increased *c-fos* expression at basal state, suggesting that fish depleted of Ugdh activity do not exhibit spontaneous seizure and are equally responsive to PTZ treatment. As noted by reviewers, *ugdh* mutant fish do not have fully-inflated swim bladders, which may contribute to their reduced locomotor activity and demise before 14-dpf (Suppl. Fig. 3e). These in vivo experiments suggest that zygotic *ugdh* depletion in zebrafish does not satisfactorily model the human disease.

**Fig. 4:**
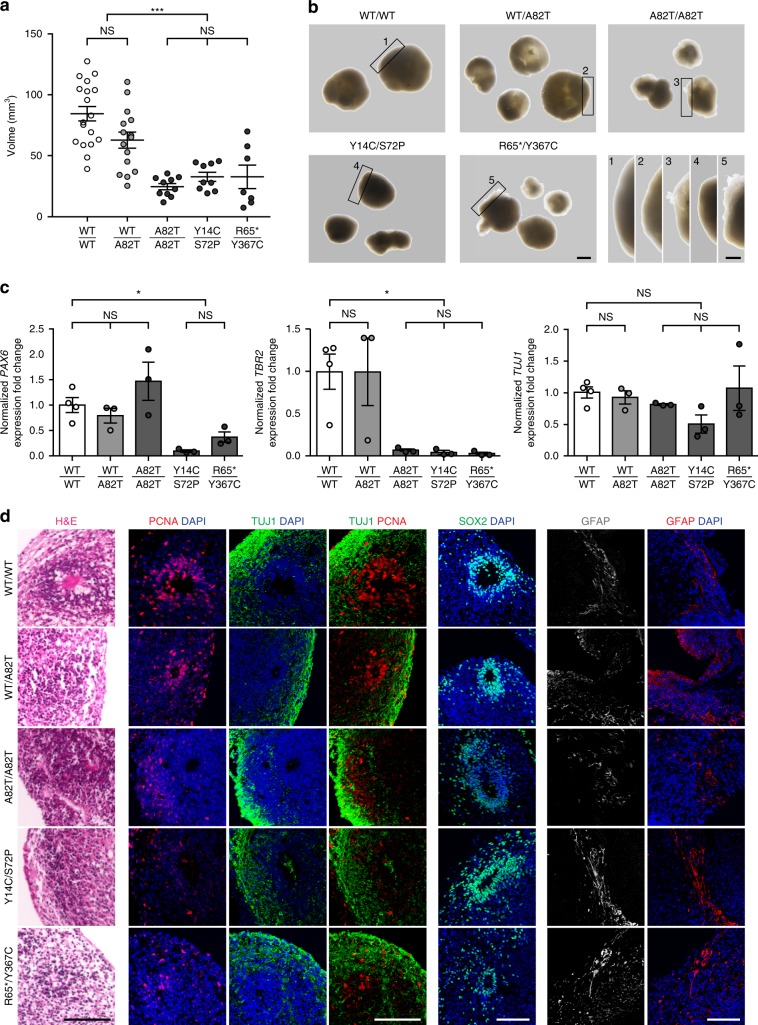
Patient-derived cerebral organoids are underdeveloped. **a** Volumes (mean ± SD) and **b** representative images (scale bar = 1 mm) of cerebral organoids derived from iPSCs from WT (*n* = 18 organoids from the same batch), unaffected parent (*UGDH* WT/A82T, *n* = 15), and patients (*UGDH* A82T/A82T (*n* = 10), Y14C/S72P (*n* = 7), and R65*/Y367C (*n* = 6) after 10 weeks of differentiation. Lower right panel: close-up views of the edges of indicated cerebral organoids. Scale bar = 500 μm. **c** RT-qPCR for neuronal differentiation markers (*PAX6*, *TBR2*, and *TUJ1*) in WT (*n* = 4 cerebral organoids), unaffected parent (WT/A82T, *n* = 3), and patients (A82T/A82T, Y14C/S72P, and R65*/Y367C, *n* = 3 each) cerebral organoids. Levels of expression are normalized to *GAPDH*. Mean ± SD fold change relative to WT is plotted. **d** Representative images of consecutive sections of cerebral organoids derived from iPSCs from WT (*N* = 5 cerebral organoids, *n* = 40 ventricle-like zones), unaffected parent (WT/A82T, *N* = 4, *n* = 15), and patients (A82T/A82T *N* = 3, *n* = 40, Y14C/S72P *N* = 4, *n* = 18, and R65*/Y367C *N* = 2, *n* = 9) stained with H&E, and immunostained with markers TUJ1/PCNA/DAPI, SOX2/DAPI, and GFAP/DAPI. Scale bar = 100 μm. **a**, **c** Asterisks indicate *p*-values of *p* < 0.05(*), *p* < 0.001(***), NS: non-significant (*p* > 0.05) as determined by ANOVA test with Bonferroni correction. **a**–**c** Cerebral organoids represented here are all from batch 2 and derived from iPSCs clone 1 for each genotype, see Suppl. Fig. 4 for more information. For graphs source data, please refer to the source data file 2.

UGDH has been extensively studied in vertebrate model organisms where its complete knockout causes embryonic lethality around gastrulation^25,26^. To address its role in the context of central nervous system (CNS) development in humans, we attempted instead to model this disease in vitro by developing cerebral organoids^27,28^ from several patients with compound heterozygous R65*/Y367C, Y14C/S72P or homozygous A82T mutations, and from a non-affected parent (WT/A82T). After 10 weeks of differentiation, the volume of cerebral organoids from patients with biallelic *UGDH* mutations was on average 50% smaller and showed rougher edges than that of WT or carrier WT/A82T cerebral organoids (Fig. 4a, b and Suppl. Fig. 4a). Quantitative reverse transcription PCR (RT-qPCR) analysis revealed decreased levels of the early and intermediate neuronal progenitors markers *PAX6* and *TBR2*, respectively, while the levels of neuronal marker *TUJ1* were unchanged (Fig. 4c and Suppl. Fig. 4b). Immunofluorescence revealed similar amounts of peripheral neurons marked by TUJ1, and astrocytes marked by GFAP while ventricular zones marked by SOX2-positive neuronal progenitors were appreciably less proliferative. This was evidenced by reduced PCNA staining in mutant cerebral organoids relative to WT and WT/A82T sections (Fig. 4d and Suppl. Fig. 4c). These results argue that reduced UGDH activity is associated with impaired neuronal development in vitro, causing atrophy of patient-derived cerebral organoids. Even though our cerebral organoid data is congruent with our patients’ phenotype and biochemistry data, replicative studies with additional WT and complete UGDH knockout lines are warranted in light of the known variability in induced pluripotent stem cells (iPSCs’) response to differentiation protocols.

To understand whether mutations in *UGDH* directly affect neuronal function, we also differentiated WT, non-affected parent (WT/A82T), and patient (Y14C/S72P) iPSCs into neuro-precursor cells (NPCs), which were subsequently matured into neurons over a period of 21 days. Using a multi-electrode array (MEA) system, and in contrast to neurons mutant for *CAMK2A*^29^, no significant differences between controls and mutant *UGDH* neurons were recorded for either the total number of spontaneous spikes or the mean firing rate (Suppl. Fig. 4d). Altogether, these in vitro experiments suggest that while UGDH is not required for proper function of isolated neurons in culture, its absence significantly affects neuronal differentiation in cerebral organoids, which may provide a powerful platform to study the pathogenesis for the new disease in vitro.

## Discussion

In this study, we described disease-causing mutations in *UGDH* in humans. These 23 coding variants represent an allelic series of germline mutations, which when inherited recessively are responsible for epileptic encephalopathy with variable degrees of developmental delay. We propose to name this novel Mendelian disease Jamuar Syndrome, a member of the early infantile epileptic encephalopathies (EIEE). The genetic, biochemical, cellular and developmental findings reveal that these *UGDH* germline mutations behave as loss-of-function alleles. This was confirmed in vitro using patient-derived cerebral organoids, which showed marked underdevelopment. In zebrafish, we found that hypomorphic *ugdh*^*I331D/I331D*^ mutant larvae did not show signs of increased seizures at baseline or after PTZ treatment. The brain-specific *UGDH* phenotype in humans may come as a surprise since in *Drosophila*, zebrafish, and mouse, complete knockout of *Ugdh* cause early and lethal gastrulation defects by hindering FGF signaling^25,30–32^. One potential explanation for this incongruity is that other proteoglycans not reliant on UGDH activity for the synthesis of UDP-GlcA or UDP-Xylose (UDP-Xyl) may be solicited and help bypass the need for UGDH during human gastrulation. Alternatively, a complete knockout of UGDH in humans may not be viable as we did not identify any homozygous or compound heterozygous truncating mutations. A search in ExAC for homozygous variants resulted in mostly synonymous or non-coding variants. Only two homozygous missense variants were detected, but no homozygous truncating mutations were seen (Suppl. Table 3). This suggests that the severity of the epileptic encephalopathy may correlate with the amount of residual UGDH activity, the extent of which may be sufficient to allow gastrulation to take place during early human embryonic stages but may be limiting for neuronal development thereafter.

As UDP-GlcA is the major product of the UGDH enzyme, it is possible that reduced levels of UDP-GlcA may trigger a cascade of secondary pathogenic events resulting in neurodevelopmental delay and encephalopathy. In support of this, is the recent demonstration that a homozygous loss-of-function mutation in the upstream enzyme UGP2 is also responsible for a severe form of developmental epileptic encephalopathy in humans^33^. UDP-GlcA is not only needed for detoxification via glucuronidation, but is also a key component of glycosaminoglycans (GAGs). UGDH deficiency might parralel other neurological diseases with defects in GAG synthesis, modification, and degradation. For example, *EXTL3* and *CHSY1* mutations, which affect heparan sulfate and chondroitin sulfate synthesis, respectively, cause developmental delay and intellectual disabilities^34,35^. Defects in heparan sulfate modification caused by *NDST1* mutations are responsible for intellectual disability associated with epilepsy^36^. Moreover, mucopolysaccaridoses, diseases caused by defects in GAG degradation, affect cognitive development^37^. In addition, proteoglycans containing GlcA derived from UDP-GlcA are major components of the extracellular matrix (ECM) and key players in neuronal development and plasticity^38^, particularly in areas important for neuronal migration^39^. In human, various psychiatric and intellectual disorders are caused by mutations in genes involved in ECM homeostasis and may be driven by neuronal migration defects^38^. The central role of UDP-GlcA may open a window for early therapeutic interventions. In plants and lower animals, including zebrafish, UDP-GlcA can be synthesized by two alternative pathways. Apart from UGDH, UDP-GlcA can be generated via the myo-inositol oxygenation pathway from glucuronic acid by glucuronokinase and UDP-glucuronic acid pyrophosphorylase^40^. If a similar route exists in humans, supplementation of glucuronate may help to enhance this alternative pathway and increase levels of UDP-GlcA levels and its essential metabolites. To this date, however, the existence of human homologs of glucuronokinase and UDP-glucuronic acid pyrophosphorylase remains to be proven.

Conservative estimates of disease frequency resulting from germline *UGDH* mutations projects a prevalence of 1:14,000,000 to 1:2,000,000 (Suppl. Note 1). Considering that developmental epileptic encephalopathies are most commonly caused by de novo dominant mutations, this estimated prevalence seems relatively frequent.

## Methods

### Ethical approval

Written informed consent was obtained from the parents of the underage patients for diagnostic procedures and next-generation sequencing, as well as for the publication of identifying facial images in Fig. 1b. The study has been approved by the local Institutional Review Board of the Medical Faculty of the University of Tübingen, Germany (vote 180/2010BO1).

### Exome sequencing

To unravel the molecular cause of the disease exome sequencing were performed at different genetic institutes using next-generation sequencing techniques according to local standard protocols. Variants were confirmed via Sanger sequencing using standard methods and chemicals (primer sequences are available on request).

*Family 1*: Exome sequencing for two affected siblings was performed on a HiSeq2500 System (Illumina, CA) after enrichment with SureSelectXT Human All Exon V5 (Agilent, Santa Clara CA). FASTQ files were imported into GENESIS (http://thegenesisprojectfoundation.org/)^12^ for further analysis using a pipeline build on BWA^41^, Picard, and FreeBayes. Variants were filtered for changes that segregated in an autosomal recessive fashion and passed the following filter criteria: (i) frequency in public databases (ExAC^10^ minor allele frequency (MAF) <0.1%), (ii) present in <5 families within GENESIS ( ∼ 4,300 exomes), (iii) conserved (PhyloP 100-way score >2 or PhastCons (100 vertebrate genomes) >0.75), (iv) CADD score >12, and (v) sufficient quality scores (Genotype Quality >75). In addition, variants had to be present in exomes from both siblings. This resulted in a list of seven variants (Suppl. Table 1), out of which only the homozygous missense variant c.131 C > T in the *UGDH* gene segregated with the third affected sibling.

*Families 2, 9, 10*: Using genomic DNA from the proband and parents, the exonic regions and flanking splice junctions of the genome were captured using the Agilent SureSelect Human All Exon V4 (50 Mb) or the Clinical Research Exome kit (Agilent Technologies, Santa Clara, CA). Massively parallel (NextGen) sequencing was done on an Illumina system with 100 bp or greater paired-end reads. Reads were aligned to human genome build GRCh37/UCSC hg19, and analyzed for sequence variants using a custom-developed analysis tool. Additional sequencing technology and variant interpretation protocol has been previously described^42^. The general assertion criteria for variant classification are publicly available on the GeneDx ClinVar submission page (http://www.ncbi.nlm.nih.gov/clinvar/submitters/26957/). After variant stratification based on population frequencies within an internal database and ExAC^10^, inheritance, in silico predictors such as Provean, Mutation Taster and CADD, GeneDX reported only the UGDH variants to be the best potentially pathogenic candidates and connected to this project via GeneMatcher entries.

*Family 3*: Samples of the oldest sibling and both parents were sequenced in context of the EUROCORES project EuroEPINOMICS-RES, for which the technical details have been reported before^43^. Briefly, the trio underwent exome sequencing at the Wellcome Trust Sanger Institute (Hinxton/Cambridge, UK). Capturing of the exome was performed using the SureSelect Human All Exon 50 Mb exome kit (Agilent). The enriched exome libraries were then sequenced on a HiSeq2000 platform (Illumina) as 75 bp paired-end reads. BWA was used to align the sequenced reads to the reference genome (hg19). De novo analysis of these data did not reveal any variants. As the younger sib later developed a similar disorder, exome sequencing was also performed locally on the second sibling: for library preparation, genomic DNA was sheared to the average size 150 bp (Covaris) and the genome libraries prepared using KAPA HTP Lib Prep Kit Illumina 96 rxns (07138008001). Exome capturing was performed using the SeqCap EZ Human Exome v3.0 capture system and the sample was sequenced on the NextSeq500 platform using NextSeq500 High-output V2 kit. Mapping to the human reference genome (Hg19) and variant calling were performed with the CLC Genomics Workbench. Subsequent annotation and filtering were executed with GenomeComb^44^ (http://genomecomb.sourceforge.net/). Exome sequencing results of the trio and the second sibling were merged and reannotated and the family was reanalyzed as a quartet. Variants were filtered based on following quality parameters: coverage >7, quality >50 and not located in homopolymers >8 or tandem repeats. Only variants present with a frequency <0.5% in control population databases ExAC^10^ and Exome Variant Server, seen <3 times in the local exome sequencing database, and with predicted impact on the encoded protein (missense, nonsense, frameshift, deletions, insertions and (essential) splice site) were retained for further analysis. Remaining variants were filtered under an autosomal recessive (homozygous or compound heterozygous) and x-linked hypothesis. In addition, heterozygous variants called in both siblings and absent in the parents were selected under a parental mosaics for which the ExAC^10^ filter was set at <2 calls. This analysis revealed only this one compound heterozygous *UGDH* variants.

*Family 4*: Parent-proband trio exomes were prepared using the SureSelect Target Enrichment System (Agilent, Santa Clara CA) and sequenced on a HiSeq2000 System (Illumina, CA). Data processing, bioinformatics pipeline (for alignment, variant calling, annotation, and genetic model filtering), and analyses were previously described^45^. The compound heterozygous rare missense alterations in the *UGDH* gene, c.214T > C and c.41A > G, were interpreted as the only candidate genetic etiology.

*Family 5*: The exome library was prepared on an ION OneTouch System and sequenced on an Ion Proton instrument (Life Technologies, Carlsbad, CA, USA) using one ION PI chip. Sequence reads were aligned to the human GRCh37/hg19. Variants were filtered for common SNPs using the NCBI’s “common and no known medical impacts” database (ClinVar), ExAC^10^, as well as an in-house database of 406 sequenced samples. Additional filters were applied to retain proband’s exonic variants that were homozygous while heterozygous in both parents. Out of 5 homozygous variants, only one missense variant c.244G > A in the *UGDH* gene was found to segregate with the disease.

*Family 6*: Exome sequencing was performed on a NextSeq 500 System (Illumina, CA USA), with a 2 × 150 bp high-output sequencing kit after enrichment with Seq Cap EZ MedExome kit (Roche, Basel Switzerland). Sequence alignment, variant calling, and variant annotation was performed by Genosplice Technology (Paris France) with BWA 0.7.12, picard-tools-1.121, GenomeAnalysisTK-2014.3-17-g0583018 and SNPEff-4.2 with additional annotations from ClinVar and HGMD. The compound heterozygous *UGDH* variants were selected to be the most promising candidates and were thus submitted to GeneMatcher.

*Families 7, 8, 16–25*: Exome sequencing was performed essentially as described before^46^ for families 7, 8, and 16–25. Target regions were enriched using the Agilent SureSelectXT Human All Exon 50 Mb Kit. Whole-exome sequencing was performed on the Illumina HiSeq platform (BGI, Copenhagen, Denmark) followed by data processing with BWA (read alignment,) and GATK (variant calling) software packages. Variants were annotated using an in-house developed pipeline. Prioritization of variants was done by an in-house designed “variant interface” and manual curation. As four families with similar phenotype shared the homozygous p.R317Q as best candidate, a GeneMatcher entry was made and the in-house database was systematically screened for other potentially pathogenic *UGDH* variants. This allowed the identification of families 18 to 25.

*Family 11*: The sequencing was performed at Claritas Genomics (Cambridge, USA). Extracted genomic DNA was amplified using the AmpliSeq system and sequenced using an IonTorrent Proton Instrument. Alignment and variant calling of the nuclear DNA was done on Proton data using Torrent Suite 4.4 Software. Nuclear variants were filtered for quality using a custom filtering tool. In addition, extracted genomic and mitochondrial DNA was also run on an Agilent Clinical Research Exome capture sequence and then sequenced using an Illumina NextSeq instrument. Alignment and variant calling on NextSeq data was performed by an implementation of GATK Best Practices Pipeline. Genomic DNA results from the two NGS runs on the proband were combined and annotated by a custom bioinformatics pipeline. Besides a heterozygous *SLC6A5* missense variant inherited from the unaffected father, the compound heterozygous *UGDH* variants were the only candidates reported that had a minor allele frequency (MAF) of < or = 0.01% that passed the laboratory’s quality metrics and were not de novo, X-linked or had biallelic variants.

*Family 12*: Isolated genomic DNA from peripheral blood leukocytes of proband and parents was captured with the Agilent Sure Select Clinical Research Exome (CRE) kit (v2). Sequencing was carried out with 150 bp paired-end reads on the Illumina HiSeq 4000. Reads alignments to the GRCh37/UCSC hg19 build were achieved using BWA (BWA-MEM v0.7.13). Variants were called using GATK (v3.7 (reference: http://www.broadinstitute.org/gatk/). Annotated Variants were filtered and prioritized using the Bench lab NGS v5.0.2 platform (Agilent technologies). The full exome analysis revealed the compound heterozygous variants in *UGDH*. The family was linked to this cohort via GeneMatcher.

*Family 13*: The exonic regions and flanking splice junctions of the genome were captured using proprietary GeneDx tools. Sequencing was performed on an Illumina system with 100 bp or greater paired-end reads. Reads were aligned to the human genome build GRCh37/UCSC hg19. A custom-developed analysis tool (Xome Analyzer) was used to call sequence variants. Sanger sequencing was used to confirm all potentially pathogenic variants identified in available family members. Additional variants not included in this report are available upon request.

*Family 14*: The patient F14-II:1 was enrolled in the ongoing “Undiagnosed Patients Program” at the Ospedale Pediatrico Bambino Gesù, Rome. Targeted enrichment (SureSelect All Exon V.4, Agilent) used genomic DNA extracted from circulating leukocytes for the affected subject and both parents, and parallel sequencing was performed using an Illumina HiSeq2000 platform, obtaining about 70 million reads. The data analysis was performed using an in-house implemented pipeline, which mainly take advantage of the Genome Analysis Toolkit (GATK V.3.7)^47^ framework, as previously reported^48,49^). The functional annotation of variants was achieved using SnpEff and dbNSFP (V.3.0)^50–52^. The functional impact of variants was analyzed by Combined Annotation Dependent Depletion (CADD) V.1.3, M-CAP V.1.0, and InterVar V.0.1.6 algorithms^11,53,54^. Two compound heterozygous private missense variants in the *UGDH* gene, c.347T > C and c.1328G > A, were interpreted as the only candidate genetic etiology.

*Family 15*: genomic DNA from of the proband and parents were enriched for exonic sequences with the SureSelect Human All Exon 50 Mb V5 Kit (Agilent Technologies, Santa Clara, California, USA). The HiSeq2500 (Illumina, San Diego, California, USA) was used to generate 125-bp paired-end runs of sequences. Reads were aligned and variant called with DNAnexus (Palo Alto, California, USA) using the reference human genome assembly hg19 (GRCh37). A mean coverage of 104x was achieved for the proband. Data analysis was preformed using an in-house bioinformatics pipeline. The compound heterozygous *UGDH* variants were selected to be the most promising candidates and were thus submitted to GeneMatcher.

### Brain magnetic resonance imaging

Magnetic resonance images (MRI) have been recorded on 1.5 or 3 Tesla scanners at the different clinical sites. Sagittal, transversal, and coronal images of the brain have been acquired with standard sequences, including T1, T2, and Flair images.

### Cell culture

Primary dermal fibroblast cultures were established from skin biopsies obtained from individuals F3-II:1, F4-II:1, F5-I:1, and F5-II:1 according to standard procedures^55^. In brief, primary fibroblasts were derived from biopsy samples and cultured in Dulbecco's modified Eagle medium (DMEM; HyClone, SH30243.01) supplemented with 10% fetal bovine serum (Biological Industries) and 2 mM l-glutamine (Biological Industries). Written informed consent of healthy probands and parents of UGDH patients were received prior to biopsy according to the ethical approvals of the local Institutional Review Boards (IRB).

### Reverse transcription (RT-PCR) and quantitative PCR

Total RNAs were extracted using the RNeasy Mini Kit (Qiagen). RNA (1 µg) was reverse transcribed using the Iscript™ complementary DNA (cDNA) Synthesis Kit (Bio-Rad). Quantitative real-time PCRs were performed using Power SYBR green master mix (Applied Biosystems) on the 7900HT Fast real-time PCR system (Applied Biosystems). qPCR primer sequences are as follows: *UGDH* (between exons 6 and 7) 5′CTTGCCCAGAGAATAAGCAG3′ and 5′CAAATTCAGAACATCCTTTTGGA3′; *β-ACTIN* 5′ATGTTTGAGACCTTCACACC3′ and 5′AGGTAGTCAGTCAGGTCCCGGCC3′; *GAPDH* 5′TGAACCACCAACTGCTTAGC3′ and 5′GGCATGGACTGTGGTCATGAG3′.

### Protein isolation and analysis

Cells were lysed using ice-cold RIPA buffer (250 mM Tris, pH: 7.5; 150 mM NaCl; 1% NP-40; 0.5% Na deoxycholate; protease inhibitors P2714 [Sigma-Aldrich, USA]). The total protein concentration of cell lysates was determined using the BCA Protein assay Kit (Thermo Fisher Scientific, USA). Sixty micrograms of total proteins were reduced in Laemeli loading buffer, denatured at 95 °C for 10 min, separated by 4–20% sodium dodecyl sulfate–polyacrylamide gel electrophoresis (Invitrogen, Germany) electrophoresis and transferred onto Immun-Blot® Low Fluorescence PVDF Membranes (BIORAD). Protein detection was performed using anti-UGDH (1:500, Sigma-Aldrich, USA, HPA036657) and anti-GAPDH (1:2000, Santa Cruz Biotechnology Inc., USA, SC 47724) antibodies. Secondary antibodies conjugated to peroxidase (1:4000, Santa Cruz Biotechnology Inc., USA) were used and blots were developed using an enhanced chemiluminescence system, Pierce™ ECL Plus (Thermo Scientific), followed by detection on autoradiographic films.

### HA quantification

HA content was compared in the culture-conditioned media from fibroblasts expressing WT or mutant UGDH using a competitive binding assay as previously described^19^. Fibroblasts were grown in 10 cm plates, 1 mL of conditioned media was aspirated from technical replicates, and cells were counted. HA concentration was interpolated from a standard curve, normalized to cell number, and plotted as mean ± SD. Statistical significance was assessed by Student’s *t*-test with at least three technical triplicates.

### Specific activity measurement of UGDH

Fibroblasts expressing WT or mutant UGDH were assayed for UGDH-specific activity essentially as previously described^56^. Fibroblasts were cultured in 15 cm plates, washed three times with cold 1x PBS, and centrifuged at 1500 rpm for 5 min. Cells were resuspended in twice the pellet volume of Lysis Buffer (50 mM Tris-HCl pH 7.4, 150 mM NaCl, 1 mM EDTA, and protease inhibitor cocktail). Samples were transferred to tubes with an equal volume of acid washed glass beads (Sigma) and lysed in the Bullet Blender 24 (Next Advance) at speed 8 for 3 min. The resulting supernatant was centrifuged at 13,000 rpm for 15 min to obtain final lysates. Enzymatic activity of the lysates (50 µg) was assayed with 1 mM UDP-glucose and 1 mM NAD^+^ in the presence or absence of 1 mM UDP-xylose, a UGDH-specific inhibitor, and monitored for changes in NADH, A_340_. Reaction rates for samples containing UDP-Xyl were subtracted from samples without UDP-Xyl to obtain UGDH-specific activity reported as [NADH] in nmol min^−1^ mg^−1^ lysate as described above. Each fibroblast cell line analyzed contained three or more technical replicates for reactions with and without UDP-Xyl plotted as mean ± SD. Statistical significance was assessed by one-way ANOVA (Prism).

### Generation and purification of *UGDH* point mutants

Point mutants of human UGDH were generated from the codon optimized *E. coli* expression construct, WT-UGDH pET28a, using polymerase chain reaction mutagenesis with appropriate primers as previously described^19,57^. Sequences were verified by Eurofins MWG Operon (Huntsville, AL). The UGDH mutant constructs were expressed in *E. coli* strain BL21(DE3) grown in 2xYT medium containing 50 mg L^−1^ kanamycin at 37 °C. At an OD_600_ of 0.6-0.8, protein expression was induced with the addition of IPTG at a final concentration of 0.5 mM, and cultures were incubated at 18 °C overnight. Cells were harvested by centrifugation and lysed by sonication. All UGDH point mutants were expressed in the soluble fraction, and enzymes were purified by affinity chromatography using a HisTrap FF column (GE Healthcare) according to the manufacturer’s protocol. The average protein yields were: ~20 mg L^−1^ for UGDH WT and T325D, ~1.5 mg L^−1^ for UGDH A82T, and ~6 mg/L for UGDH A44V. Purified protein was dialyzed against 20 mM Tris-HCl pH 7.4 containing 1 mM dithiothreitol (DTT), concentrated, flash frozen in liquid nitrogen, and stored at –80 °C.

### Analytical gel filtration

Purified recombinant UGDH WT and all point mutants were analyzed by size exclusion chromatography as previously described^57^. All samples were centrifuged prior to loading. Each apoprotein sample was injected into a 250 µL loop and separated by FPLC in 1x PBS containing 1 mM DTT at a flow rate of 0.5 mL min^−1^ on a Superdex 300 10/200 GL gel filtration column (GE Healthcare). Elution was monitored by A_280_ and plotted to compare alterations in oligomeric state.

### Trypsin susceptibility assay

Purified recombinant WT-UGDH and all point mutants were assessed by limited trypsin proteolysis as previously described^19^. UGDH WT, A44V (10 µg), and A82T (14.2 µg) were digested with 10 ng trypsin in 1x PBS pH 7.4 for 2.5 h at room temperature in the absence or presence of 1 mM UDP-glucose, 1 mM UDP-glucuronate, 5 mM NAD^+^, 5 mM NADH, or combinations that yielded abortive and productive ternary complexes. Samples were analyzed by western blot probed for UGDH as previously described^58^.

### Thermal stability measurement

Recombinant UGDH WT and A44V protein were assessed for thermal stability as previously described^19^ with minor alterations. All samples of UGDH WT and A44V (~15 µg) were incubated in 1x PBS and Sypro Orange dye (Invitrogen; 1:500 dilution) in the absence or presence of 1 mM UDP-glucose, 1 mM UDP-glucuronate, 5 mM NAD^+^, 5 mM NADH, or in combinations that yielded abortive and productive ternary complexes. Samples were handled at room temperature and transferred to an iCycler MyiQ thermocycler (Bio-Rad) for incremental thermal denaturation. *T*_m_ was plotted as the mean ± SD for seven replicates. Statistical significance was assessed by two-way ANOVA (Prism).

### Saturating enzymatic activity and kinetic characterization

Enzyme activity of recombinant UGDH WT and all point mutants was characterized as described previously^57^ with minor alterations. Enzymatic activity was calculated by NADH turnover using the NADH extinction coefficient of 6220 M^−1^ cm^−1^. UGDH A82T activity was converted to [NADH] in nmol min^−1^ mg^−1^ UGDH and subsequently normalized to the fractional purity of UGDH in the sample preparation. Samples were run in triplicate and statistical significance was determined using Student’s *t*-test. Michaelis rate constants, *K*_m_ and *V*_max_, were determined for UGDH WT and A44V as previously described using a 96-well plate assay to measure the change in NADH (A_340_) with respect to both the substrate, UDP-glucose, and cofactor, NAD^+^.

### iPSCs reprogramming

WT, WT/A82T, and A82T/A82T fibroblasts were reprogrammed using the CytoTune™-iPS 2.0 Sendai Reprogramming Kit (Thermo Fisher Scientific, A16517) in accordance with the manufacturer’s instructions. Briefly, fibroblasts were transduced and plated after 7 days onto Matrigel Basement Membrane Matrix (Corning, 354234) in mTeSR1 medium (STEMCELL Technologies, 85850). iPSC colonies were picked between days 17–28 and maintained in Matrigel Basement Membrane Matrix and mTeSR1 for expansion. R393W/A410S, R65*/Y367C, Y14C/S72P fibroblasts were reprogrammed using the ReproRNA™-OKSGM kit (Stemcell Technologies, 05930) in accordance with the manufacturer’s instructions. Briefly, fibroblasts were plated onto Matrigel Basement Membrane Matrix (Corning, 354234) and transfected with ReproRNA™-OKSGM cocktail. Puromycin selection was carried out 1 day after transfection. iPSC colonies were picked between 20 and 28 days after transfection and maintained in Matrigel Basement Membrane Matrix and mTeSR1 for expansion. Between 1 and 3 clones per genotype were maintained for further experiments.

### Neuronal and cerebral organoid differentiation

Neuronal and cerebral organoid differentiation was performed as previously described^27^. Briefly, on day 0 of organoid culture, iPSCs were dissociated by accutase (STEMCELL Technologies, 07920) treatment to generate single cells. In total, 9000 cells were then plated per well of an ultra-low-binding 96-well plate (Corning) in MEDI medium [Knockout SR 20% (Thermo Fisher scientific, 10828-028), l-glutamine 2 mM (Thermo Fisher scientific, 200 mM, 25030-081), Non-essential amino-acid (NEAA) 1 × (Thermo Fisher scientific, 100× , 11140-050), sodium pyruvate 1× (Thermo Fisher scientific, 100 mM, 11360-070), and β-mercaptoethanol 1/1000 in neurobasal medium (Thermo Fisher scientific, A2477-501)] complemented with 10 µM Rho-associated protein kinase (ROCK) inhibitor (STEMCELL technology, Y-27632) and 0.05% polyvinyl alcohol. Embryoid bodies were fed with this medium every other day for 6 days. Then, MEDI medium was replaced with NIM-I medium [N2 1× (Thermo Fisher scientific, 100× , 17502-048), l-glutamine 2 mM, NEAA 1× , and heparin 1 µg mL^−1^ (Sigma-Aldrich, H3149) in neurobasal medium] every other day for 6 additional days for the neuroepithelial tissues induction. On day 11, tissues exhibiting neural ectoderm were transferred to droplets of cold Matrigel (BD Biosciences) on a sheet of Parafilm with dimples in a low-adhesion 6-well plate (Corning). These droplets were allowed to gel at 37 °C and were subsequently removed from the Parafilm and grown in differentiation medium Dif-M [N2 0.5 × , B27 without vitamin A 1× (Thermo Fisher scientific, 50 × , 12587-010), l-glutamine 2 mM, NEAA 1× , and β-mercaptoethanol 1/1000 in neurobasal medium] for 4 days. On day 15, the medium was replaced by Dif-M II medium [N2 0.5× , B27 1× (Thermo Fisher scientific, 50× , 17504-001), l-glutamine 2 mM, NEAA 1× , and β-mercaptoethanol 1/1000 in neurobasal medium], and the plates are placed on a horizontal shaker rotating at 85 rpm. Cerebral organoids were grown up to 10 weeks with 75% of the medium changed weekly.

### Histology and immunofluorescence of organoids

Cerebral organoids were fixed in 4% paraformaldehyde (PFA) overnight at 4 °C, then washed in PBS for 10 min and dehydrated by incubations in Ethanol (70%, 95% then 100%) for 1 h at 4 °C followed by two times 1 h incubation with Xylene 100% at room temperature. The cerebral organoids were then embedded in Paraplast Plus (Leica, 39602004) and sectioned at 30 µm. Tissue sections were stained with Haematoxylin and Eosin (H&E) or used for immunostaining. For immunofluorescence, antigen retrieval was performed by using the Antigen Retriever buffer (Citrate Buffer pH 6.0, Sigma, C 9999, 10× ). Sections were then blocked and permeabilized in blocking buffer (0.5% Triton X-100 and 1% BSA in PBS) for 20 min. Sections were then incubated with primary antibodies in blocking buffer at the following dilutions: SOX2 (mouse, R&D systems, MAB2018, 1:200), TUJ1 (mouse, Biolegend MMS-435P, 1:3000), GFAP (Rabbit, Dako Z0334, 1/2500), PCNA (Rabbit, abcam ab18197, 1 µg mL^−1^). For visualization, an antibody anti-mouse immunoglobulin G (anti–mouse IgG) Alexa Fluor 594 conjugate (Invitrogen, Molecular Probes) and an anti-rabbit IgG Alexa Fluor 488 conjugate (Invitrogen, Molecular Probes) was applied. DNA was stained by DAPI (1/500) and sections were mounted in ProLong Diamond Antifade mountant (Thermo Fisher Scientific, P36965). Images were collected by using an Olympus FV3000 RS with a 20x objective.

### RT and RT-qPCR

Total RNA of individual cerebral organoids was extracted using the RNeasy Mini Kit (Qiagen, 74104). Total RNA (0.5 µg) was reverse transcribed using the Iscript™ cDNA Synthesis Kit (Bio-Rad, 1708891). Real-time quantitative PCRs were performed using the Power SYBR green master mix (Applied Biosystems, 4309155) on the 7900HT Fast real-time PCR system (Applied Biosystems). qPCR primers were as previously described:^59^ *PAX6* 5′CCGTGTGCCTCAACCGTA3′ and 5′CACGGTTTACTGGGTCTGG3′; *TBR2* 5′AAATGGGTGACCTGTGGCAAAGC3′ and 5′CTCCTGTCTCATCCAGTGGGAA3′; *TUJ1* 5′TCAGCGTCTACTACAACGAGGC3′ and 5′GCCTGAAGAGATGTCCAAAGGC3′.

### Neural induction

Neural induction was carried out by dual SMAD inhibition^60^. iPSC were first dissociated into single cells and seeded in low attachment 96-well u-bottom plate at a density of 10,000 cells/well in Neural Induction Medium [DMEM/F12 (Thermo Fisher Scientific, 10565-018) supplemented with B27 (Thermo Fisher Scientific, 17504044), N2 (Thermo Fisher Scientific, 17505048), 0.2 mM NEAA (Thermo Fisher Scientific, 11140-050), 100 nM LDN 193189 (STEMCELL Technologies, 72148), 10 µM SB431542 (STEMCELL Technologies, 72234) and 10 µM Y-27632 (STEMCELL Technologies, 72304)]. After 6 days, cells were attached onto matrigel coated-plate in Neural Expansion Medium [DMEM/F12 (Thermo Fisher Scientific, 10565-018) supplemented with B27 (Thermo Fisher Scientific, 17504044), N2 (Thermo Fisher Scientific, 17505048), 0.2 mM NEAA (Thermo Fisher Scientific, 11140-050) and 20 ng mL^−1^ βFGF (Stemgent, 03-0002)]. After 3–6 days, rosette structures were manually cut out and expanded as suspension culture in neural expansion medium.

### Neuronal differentiation

iPSCs-derived Neuro-Precursor Cells (NPCs) were differentiated into neurons for 21 days using a previously published protocol^61^ Briefly, NPCs were plated at a density of 50,000 cells cm^−2^ in a poly-l-ornithine and laminin-coated plates, cultured in N2B27 medium supplemented BDNF (20 ng mL^−1^), GDNF (20 ng mL^−1^), cAMP (N6,2′-O-dibutyryladenosine 3′,5′-cyclic monophosphate; Sigma; 0.3 mM) and ascorbic acid (0.2 mM).

### Multi-electrode Array (MEA) recordings

Neurons on day 21 were dissociated and replated on 0.1 polyethylenimine (Sigma)-coated 48-well MEA plates (Axion Biosystems) in BrainPhys media supplemented with BDNF, GDNF, cAMP, and ascorbic acid as previously described^62^. Spontaneous neuronal activity was observed and recorded at 37^o^ C for 5 min every 2–3 days using the Maestro MEA System (Axion Biosystem).

### Zebrafish strains and maintenance

All zebrafish husbandry procedures were performed in compliance with the Singapore National Advisory Committee on Laboratory Animal Research Guidelines from the Institutional Animal Care and Use Committee (IACUC, IACUC number 161172). The *jek ugdh*^*m151*^ mutant line^63^ was used for this study and was maintained in the AB background. All embryos were raised in egg water (5 mM NaCl, 0.17 mM KCl, 0.33 mM CaCl_**2**_, 0.33 mM MgSO_**4**_).

### Genomic DNA extraction

Genomic DNA from larvae used for the locomotion assay was extracted by larval fin clipping as previously described^64^. Briefly, microscopic caudal fin slices of 3-dpf larvae were sectioned under a stereomicroscope. The fins were then digested with proteinase K (Invitrogen, 25530049) overnight at 65 °C, and the supernatant was used as a source of genomic DNA for genotyping. In 7-dpf larvae, heads were used for *c-fos* qPCR analysis, while the rest of the bodies was used for genotyping.

### Genotyping of zebrafish

Genotyping was performed via nested PCR to amplify the exon 7 of *ugdh*, and restriction fragment length polymorphism as described previously^63^. The first PCR was performed with the following primers, 5′-TGTATGTGCAGGTGATTGACA-3′ and 5′-TGTAGGTCACAGGTTTTTGACA-3′. PCR products were cleaned-up by Exonuclease I (New England Biolabs, M0293L) and FastAP Thermosensitive Alkaline Phosphatase (Thermo Fisher, EF0651) treatment, and were then used as templates for a second PCR with the following primers: 5′-GACATGAATGAATATCAGAGAAAGAG-3′ and 5′-AGGAGAAACCCAACAACGC-3′. These PCR products were digested with the *MluI* enzyme (New England Biolabs, R3198L) that only cuts when the mutation is present.

### *c-fos* qPCR experiments

7-dpf larvae were first incubated with increasing concentrations (up to 15 mM) of PTZ (Sigma, P6500) for 45 min and were then decapitated. The bodies were used for genotyping and 20 larvae heads of the same genotype were pooled together for total RNA extraction using the RNeasy Mini Kit (Qiagen, 74104). Total RNA (0.5 µg) was reverse transcribed using the Iscript™ cDNA Synthesis Kit (Bio-Rad, 1708891). Real-time quantitative PCRs were performed using the Power SYBR green master mix (Applied Biosystems, 4309155) on the 7900HT Fast real-time PCR system (Applied Biosystems). qPCR primers were as follows: *c-fos*^23^ 5′-AACTGTCACGGCGATCTCTT-3′ and 5′-GCAGGCATGTATGGTTCAGA-3′; *gapdh* 5′-GTGGAGTCTACTGGTGTCTT-3′ and 5′-GTGCAGGAGGCATTGCTTAC-3′.

### Twenty-four-well locomotion and convulsion test assay

A Basler Ace (acA1300-200um; 1280 × 1024) camera was used to acquire videos of larval zebrafish at 50 fps. A custom hardware setup was designed to acquire full frame videos of 24-well flat bottom plates using a 25 mm lens attachment to the camera placed 65 cm above the plate. The 24-well plates were backlit using a white light LED lightbox that delivered uniform lighting across the entire field. 7-dpf larval fish were placed individually in each well in 500 µl of egg water and acclimated to the setup for 10 min. Then, 500 µl of either egg water (negative control) or PTZ 30 mM (15 mM final concentration, Sigma, P6500) was added to the well. Three videos of 2 min each were acquired for a duration of 10 min for each condition tested. Fish locomotion was tracked online during video recording on a custom written software in LabView (www.critta.org). Analysis of locomotion was automated and performed blind offline after each experiment using custom written Python scripts (available on Github: https://github.com/mechunderlyingbehavior/24-Well-Larval-Locomotion.git). In this assay, low and high speed were defined as average speeds of 0 to 8 mm/s and >8 mm s^−1^, respectively, based on a recent publication measuring convulsive-induced speed changes in zebrafish larvae^65^.

## Supplementary information

Supplementary Information

Supplementary Data 1

Description of Additional Supplementary Files

## Data Availability

The data that support the findings of this study are available within the paper and its supplementary information files. All identified variants have been deposited in the ClinVar database (https://www.ncbi.nlm.nih.gov/clinvar/) under the name “UGDH001”. Whole dataset from Family F3 was already published^66^ and deposited in the European Genome-phenome Archive, accession numbers EGAS00001000190, EGAS00001000386, and EGAS00001000048. Whole datasets from Families F5 and F15 are available upon request from the corresponding authors. Consent restrictions preclude deposition of sequencing data of the other families, however, specific information (e.g., secondary variants etc., but not full datasets) can be obtained upon request from the corresponding authors. Lists of primers and antibodies are in Supplementary Tables 4 and 5, respectively. The source data underlying Fig. 3a–f, Fig. 4a, and c, Suppl. Fig. 3a–d, Suppl. Fig. 4a, b, and d are provided as a Source Data file 1 (for gels) and 2 (for graphs).

## Source data

Source Data

## Code availability

The custom written Python scripts used to track fish locomotion is available following this link: https://github.com/mechunderlyingbehavior/24-Well-Larval-Locomotion.git.
