# Supplementary material for: Loss-of-function mutations in UDP-Glucose 6-Dehydrogenase cause recessive developmental epileptic encephalopathy
Source: Nat Commun. 2020 Jan 30;11:595. doi: 10.1038/s41467-020-14360-7 (PMC6992768; doi:10.1038/s41467-020-14360-7)
Supplement: Supplementary file 1 — Supplementary Information [file 41467_2020_14360_MOESM1_ESM.pdf]

Loss-of-function Mutations in *UDP-Glucose 6-Dehydrogenase* Cause Recessive Developmental Epileptic Encephalopathy

Supplementary Information

H. Hengel & C. Bosso-Lefèvre *et al.*, 2020

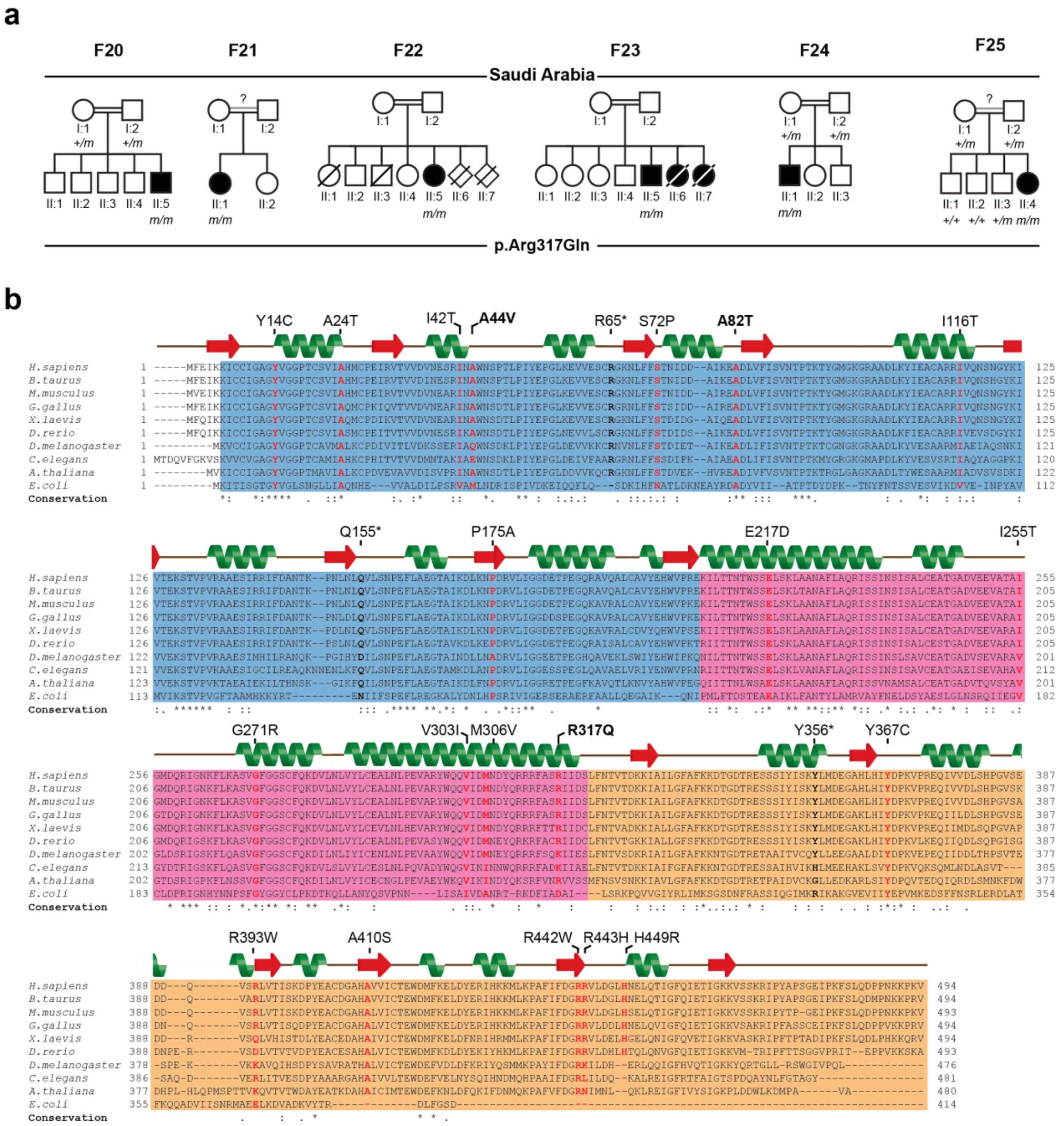

Supplementary Figure 1

Supplementary Figure 1: UGDH mutations affect highly conserved residues. (**a**) Pedigrees of families F20 to F25 from Saudi Arabia segregating autosomal recessive developmental epileptic encephalopathy. Filled black symbols, affected individuals. Crossed symbols, deceased individual. Mutations in UGDH protein are presented below pedigrees. Homozygous mutations are presented in bold (*m* in the pedigrees). Healthy siblings that could be sequenced are either homozygous WT (F25-II:1 and II:2) or heterozygous (F25-II:3) for the p.Arg317Gln mutation. (**b**) UGDH proteins alignment (using the CLUSTAL OMEGA software[1,2]) shows the conservation of the 22 affected amino acids found in 26 patients. The conservation of the mutated residues is shown in red (missense) and black (nonsense). Mutations found in a homozygous state are shown in bold. NAD-binding (blue), UDP-binding (orange) and central (pink) domains are highlighted. The secondary structure of UGDH is presented above the alignment as β-sheets (red arrows) and α-helices (green spirals).

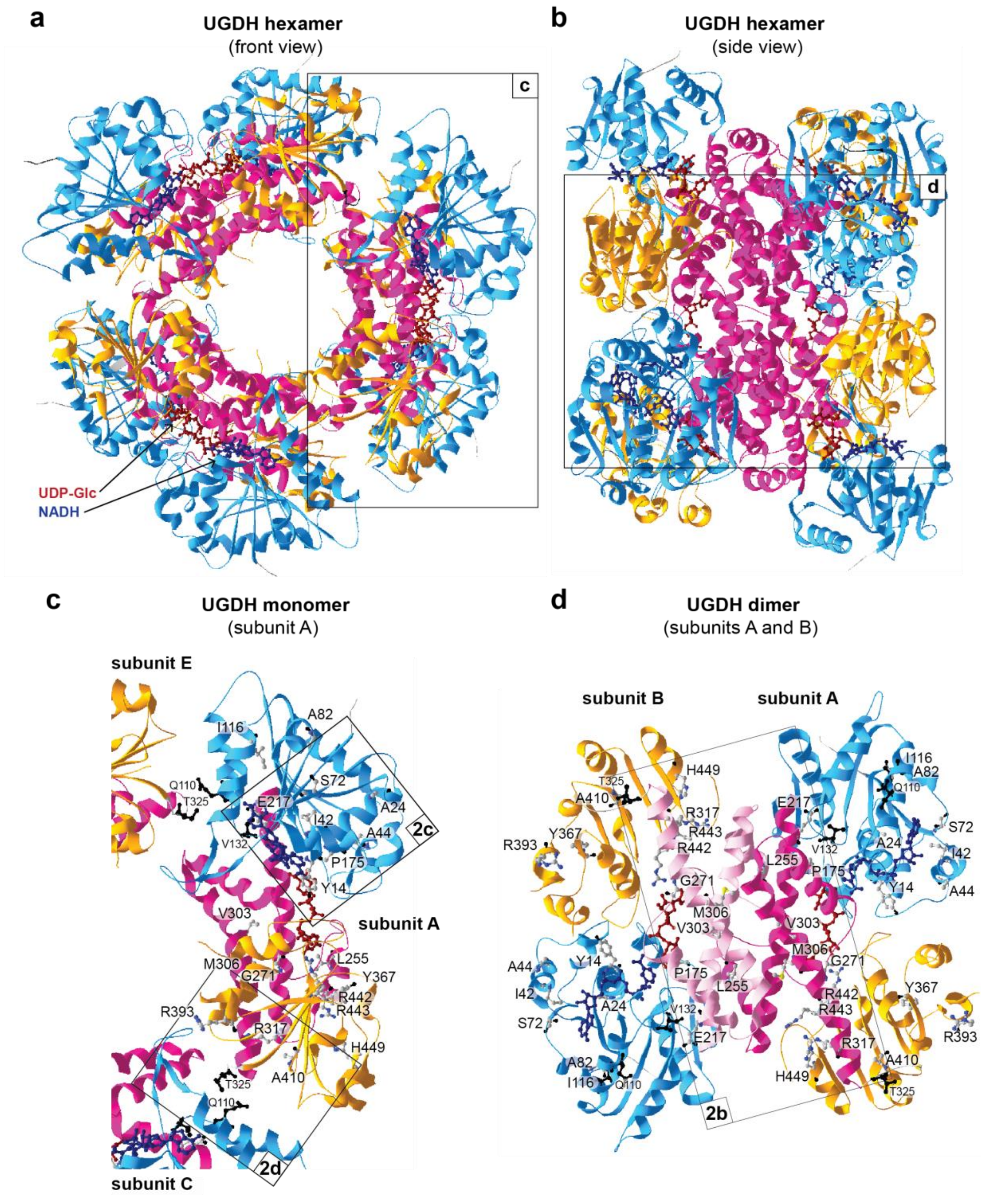

Supplementary Figure 2

Supplementary Figure 2: Mutations in UGDH enzyme possibly alter its oligomerization. Front (**a**) and side (**b**) views of the ribbon diagram of the UGDH hexamer bound to UDP-Glc and NADH. (**c-d**) Two close-up views of the ribbon diagrams of the UGDH protein bound to UDP-Glc and NADH as a monomer (**d**, subunit A; subunits C and E are shown at their interface with subunit A) and a dimer (**e**, subunits A and B). Residues carrying missense mutations in the patients are highlighted as 3D backbone. Residues Q110 and T325 known to interact together for dimer formation[3]; and residue V132 (important for hexamerization[3]) are highlighted in black backbone. Inserts 2b, 2c and 2d correspond to the close-up views shown in Figure 2b, c and d, respectively. In all the structures, NAD-binding (blue), central (light/dark pink), and UDP-binding (orange) domains are shown. UDP-Glc (dark red) and NADH (midnight blue) are represented as colored carbon backbones. Adapted from PDB code 2Q3E[4] using the Swiss-Pdb Viewer software[5].

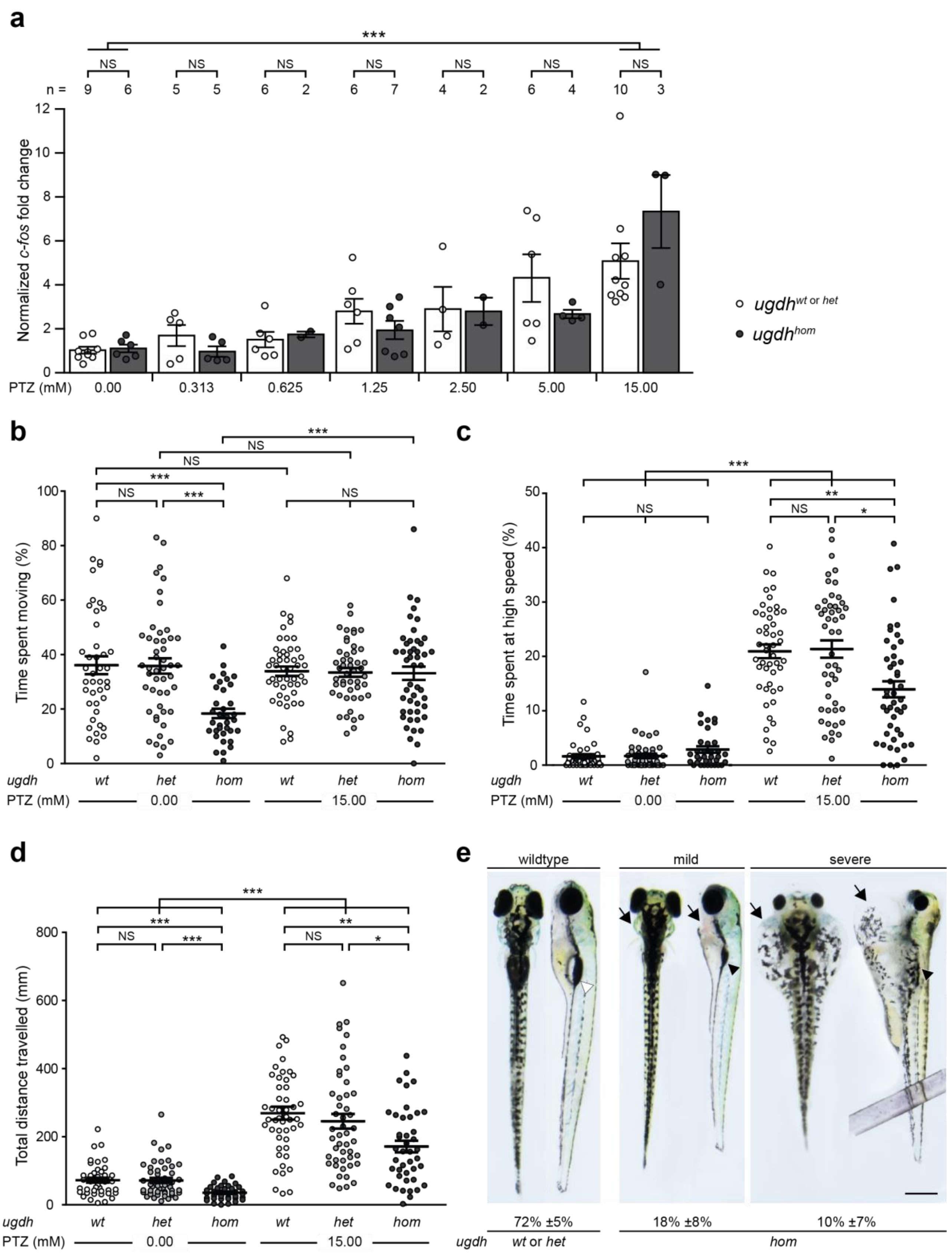

Supplementary Figure 3

Supplementary Figure 3: Mutant *ugdh*$^{I331D/I331D}$ zebrafish larvae are not more susceptible to seizures. (**a**) qPCR analysis for endogenous *c-fos* expression in the heads of 7-dpf (days post fertilisation) old larvae incubated with increasing PTZ concentration (from 0 to 15 mM as indicated below the x axis). *n* biological replicates of 20 heads each is indicated above the graph. (**b-d**) Locomotion assay in 7-dpf old larvae without (*n*= 42 wt, *n*= 48 heterozygous, and *n*= 36 homozygous larvae) or with PTZ 15 mM *(n= 48*, *n= 48*, and *n= 42,* respectively). Plotted are (**b**) the percentage of time that larvae spent moving, (**c**) the percentage of time spent at high speed, and (**d**) their total distance travelled in a user-defined area during a total recorded time of 6 minutes. (**a-d**) Data are mean ±s.e.m. NS, not significant, *P<0.05, **P<0.01, ***P<0.001, one-way ANOVA with Bonferroni's correction. (**e**) Dorsal and lateral views of control and homozygous larvae at 7-dpf showing wild type, mild (slight pericardial edema, non-inflated swim bladder), and severe (extensive pericardial edema, non-inflated swim bladder) phenotypes. The proportion of each observed phenotype from *ugdh*$^{wt/i331d}$ incrosses is indicated below the pictures as percentage. Data are mean ±s.e.m from *n* = 10 incrosses with a total of 1877 larvae. Pericardial edema (black arrow), inflated swim bladder (white arrow head), and non-inflated swim bladder (black arrow heads) are indicated. Scale bar: 100 µm (**a-e**) Abbreviations: *wt*: *ugdh*$^{wt/wt}$ wild type larvae, *het*: *ugdh*$^{wt/i331d}$ heterozygous larvae and *hom*: *ugdh*$^{i331d/i331d}$ homozygous larvae. For graphs source data, please refer to the source data file 2.

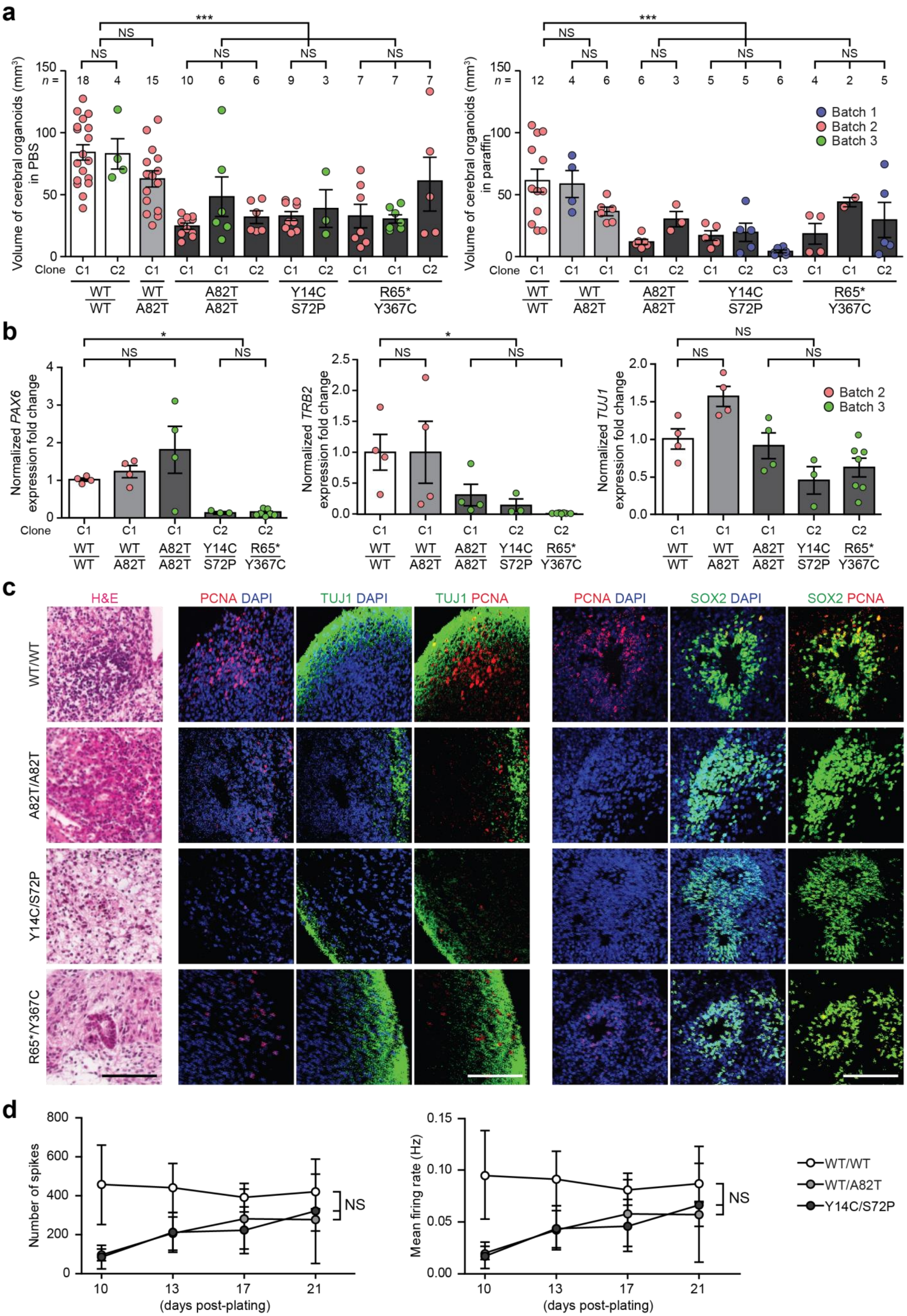

Supplementary Figure 4

H. Hengel & C. Bosso-Lefèvre *et al.*, 2020
Supplementary information

Supplementary Figure 4: Patient-derived cerebral organoids are underdeveloped while isolated neurons have normal activity (relative to Fig. 4). (**a**) Volumes of cerebral organoids derived from iPSCs from WT, unaffected parent (*UGDH* WT/A82T), and patients (*UGDH* A82T/A82T, Y14C/S72P, and R65*/Y367C) after 10 weeks of differentiation from 3 independent batches (as indicated). Volumes of cerebral organoids in PBS (left panel, batches 2 and 3) and in paraffin (right panel, batches 1 and 2) are shown. The iPSCs clone number used to derive the cerebral organoids (c), and the number of cerebral organoids analyzed (*n*) are indicated. Mean ± SD is plotted. The data presenting in **Fig. 4a** are also included. (**b**) RT-qPCR analysis for neuronal differentiation markers (*PAX6*, *TBR2* and *TUJ1*) in cerebral organoids derived from iPSCs from WT (n= 4 cerebral organoids), unaffected parent (WT/A82T, n= 4), and patients (A82T/A82T (n= 4), Y14C/S72P (n= 3), and R65*/Y367C (n= 7). Data are mean ± SD fold change relative to WT, and normalized to *GAPDH*. The iPSCs clone numbers used to derive the cerebral organoids (c), and the 2 independent batches of cerebral organoids are indicated. The data from batch 2 are also reported in **Fig. 4c**. (**c**) Representative images of consecutive sections of cerebral organoids derived from clone 2 iPSCs from WT, and patients (A82T/A82T, Y14C/S72P, and R65*/Y367C) stained with H&E, and immunostained with markers TUJ1/PCNA/DAPI, SOX2/PCNA/DAPI. Scale bar = 100 µm. (**d**) Graphical representation of the number of neuron-evoked spikes and mean firing rate detected by multi-electrode arrays (n= 6 per line per time-point) of NPCs-derived neurons. Mean ± SD is plotted. (**a, b, d**) Asterisks indicate p-values of p<0.05(*), p<0.01(**), p<0.001(***), NS: non-significant (p>0.05) as determined by ANOVA test with Bonferroni correction (**a**, **c**) or with Tukey's multiple correction (**d**). For graphs source data, please refer to the source data file 2.

Chr.	Start	End	Ref	Alt	status	Variant	ExAC	SIFT [0;1]	Polyphen2 (HumDiv) [0;1]	CADD [10;60]	GERP++ [-12.3;6.17]	phyloP (100way)	Comment
4	39523002	39523002	G	A	hom	UGDH : NM_003359 ; exon2 : c.131C>T ; p.A44V	1.65E-05	0.01	0.871	33	6.07	9.434	segregates
1	230824270	230824270	G	T	hom	COG2 : NM_007357 ; exon15 : c.1756G>T ; p.A586S	.	0.07	1	32	5.67	9.071	does not segregate
19	1036553	1036553	G	T	hom	CNN2 : NM_004368 ; exon6 : c.646G>T ; p.A216S	.	0.03	0.994	19.7	4.04	9.34	does not segregate
22	30500401	30500401	G	A	hom	HORMAD2 : NM_001329457 ; exon6 : c.269G>A ; p.C90Y	9.95E-05	0.01	1	18.35	5.19	3.859	does not segregate
1	229600595	229600595	G	A	hom	NUP133 : NM_018230 ; exon18 : c.2327C>T ; p.T776M	3.30E-05	0.12	0.96	17.32	5.27	5.412	does not segregate
1	231396281	231396281	A	G	hom	GNPAT : NM_014236 ; exon3 : c.290A>G ; p.D97G	.	0.08	0.653	17.15	4.83	7.192	does not segregate
1	215793416	215793416	A	G	hom	KCTD3 : NM_016121 ; exon18 : c.1904A>G ; p.H635R	8.62E-06	0.46	0	12.3	5.77	3.253	does not segregate

Supplementary Table 1: Variant list after filtering for homozygous or compound heterozygous variants in exomes from patient F1-II:3 and F1-II:6. Annotations were obtained using ANNOVAR[6]. CADD scores are PHRED-scaled. Abbreviations: Chr.: chromosome; ".": variant not found in ExAC[7]; Ref: reference; Alt: alternative; ND: not determined; hom: homozygous.

Family	Chr.	Start	End	Ref	Alt	Variant	EVS6500	ExAC	gnomAD	SIFT	Polyphen2 (HumDiv)	CADD	GERP++	phyloP (100way)	ΔΔG (Kcal/mol)
F7	4	39505523	39505523	T	C	c.1346A>G;p.H449R	.	.	.	0.78	0.006	15.11	5.62	7.961	-1.05
F12, F14, F15	4	39505541	39505541	C	T	c.1328G>A;p.R443H	.	.	.	0.03	0.999	35	5.62	7.741	-1.28
F15	4	39505545	39505545	G	A	c.1324C>T;p.R442W	.	.	4.06E-06	0	1	23.7	4.78	4.839	-0.856
F3	4	39506072	39506072	C	A	c.1228G>T;p.A410S	.	1.66E-05	8.12E-06	0	0.999	28.2	4.78	7.411	-2.4
F3	4	39506123	39506123	G	A	c.1177C>T;p.R393W	.	8.94E-06	1.63E-05	0	0.993	20.5	4.75	3.811	-0.5
F6	4	39506928	39506928	T	C	c.1100A>G;p.Y367C	.	.	.	0	1	23.2	6.03	7.425	-1.8
F8	4	39506960	39506960	A	C	c.1068T>G;p.Y356*	.	.	.	0.15	n/a	36	-0.679	0.404	n/a
**F16-F25**	4	39507325	39507325	C	T	c.950G>A;p.R317Q	.	5.77E-05	4.89E-05	0.02	0.982	20.9	4.87	4.688	-1.23
F2	4	39507359	39507359	T	C	c.916A>G;p.M306V	.	.	.	0.64	0.193	15.01	5.07	7.698	-1.13
F12	4	39507368	39507368	C	T	c.907G>A;p.V303I	.	.	.	0.02	0.961	23.1	4.98	7.487	-1.07
F10	4	39511380	39511380	C	G	c.811G>C;p.G271R	.	.	.	0	1	29.9	5.51	7.167	-0.42
F10	4	39511427	39511427	A	G	c.764T>C;p.I255T	.	.	8.15E-06	0	0.999	23.4	5.51	8.569	-3.21
F11	4	39511985	39511985	C	G	c.651G>C;p.E217D	.	.	.	0	1	18.34	1.39	0.564	-2.31
F9	4	39512113	39512113	G	C	c.523C>G;p.P175A	.	.	.	0.08	0.997	21.7	5.12	9.196	-1.64
F2, F7	4	39512283	39512283	G	A	c.463C>T;p.Q155*	.	.	4.06E-06	0.14	n/a	36	5.12	9.137	n/a
F14	4	39512399	39512399	A	G	c.374T>C;p.I116T	.	.	.	0	0.998	26.1	5.95	8.642	-3.24
**F5**	4	39515723	39515723	C	T	c.244G>A;p.A82T	.	.	.	0.03	0.999	23.4	3.93	6.915	-1.83
F4	4	39515753	39515753	A	C	c.214T>G;p.S72A	.	.	.	0	0.949	28.8	5.66	8.263	-0.35
F6, F13	4	39515774	39515774	G	A	c.193C>T;p.R65*	.	9.96E-05	7.43E-05	1	n/a	24.6	1.78	2.743	n/a
**F1**, F11, F13	4	39523002	39523002	G	A	c.131C>T;p.A44V	.	1.65E-05	2.05E-05	0.01	0.871	33	6.07	9.434	-0.27
F8	4	39523008	39523008	A	G	c.125T>C;p.I42T	.	.	.	0	0.999	26.5	6.07	8.923	-2.76
F9	4	39523063	39523063	C	T	c.70G>A;p.A24T	.	.	.	0	1	36	6.07	7.456	-2.07
F4	4	39523092	39523092	T	C	c.41A>G;p.Y14C	7.70E-05	8.24E-06	4.10E-06	0	1	26.8	6.07	7.665	-0.18

Supplementary Table 2: Genetic characteristics of identified UGDH mutations and their predicted effect on the protein stability. Families carrying homozygous mutations are depicted in bold. All cDNA positions refer to transcript NM_003359. Annotations were obtained using ANNOVAR[6]. CADD scores are PHRED-scaled. $\Delta\Delta G$ is the predicted stability change expressed as variation in Gibbs Free Energy (Kcal mol^{-1}) with negative values denoting destabilizing mutations, as determined by DUET software[8]. Abbreviations: Chr: chromosome; ".": variant not found in database; Ref: reference; Alt: alternative; n/a: not applicable.

Chr.	Position	Ref	Alt	Variant	HMZ	MAF in ExAC	SIFT	PolyPhen-2 (HumDiv)	CADD	GERP++	phyloP (100 way)	Comment
4	39501722	CAAA	C	c.*38_*40delTTT	2319	4.38E-01	.	.	.	.	.	3' UTR variant
4	39501722	CAA	C	c.*39_*40delTT	111	1.27E-01	.	.	.	.	.	3' UTR variant
4	39501722	CA	C	c.*40delT	37	9.19E-02	.	.	.	.	.	3' UTR variant
4	39501722	CAAAA	C	c.*37_*40delTTTT	8	1.35E-01	.	.	.	.	.	3' UTR variant
4	39528752	G	A	c.-8+151C>T	2907	7.30E-01	.	.	.	.	.	intronic variant
4	39511359	T	C	c.811+21A>G	2685	1.73E-01	.	.	.	.	.	intronic variant
4	39510175	G	A	c.906+11C>T	2659	1.72E-01	.	.	.	.	.	intronic variant
4	39515655	T	C	c.264+48A>G	2391	1.67E-01	.	.	.	.	.	intronic variant
4	39510329	C	T	c.812-49G>A	1350	1.43E-01	.	.	.	.	.	intronic variant
4	39506144	C	T	c.1172-16G>A	1047	1.06E-01	.	.	.	.	.	intronic variant
4	39515853	T	C	c.163-49A>G	51	2.33E-02	.	.	.	.	.	intronic variant
4	39506997	G	GA	c.1038-8dupT	4	8.69E-03	.	.	.	.	.	intronic variant
4	39505456	C	G	c.1374+39G>C	1	2.01E-05	.	.	.	.	.	intronic variant
4	39505643	C	T	c.1264-38G>A	1	2.97E-04	.	.	.	.	.	intronic variant
4	39507206	A	C	c.1037+32T>G	1	4.40E-04	.	.	.	.	.	intronic variant
4	39506052	C	G	c.1248G>C;p.E416D	1	1.21E-03	0.05	0.511	13.66	1.48	0.594	Missense variant. Prediction scores and MAF are not suggestive for a disease causing variant
4	39506938	G	C	c.1090C>G;p.L364V	1	2.23E-04	0.63	0.062	14.31	6.03	7.293	Missense variant. Prediction scores and MAF are not suggestive for a disease causing variant
4	39506997	GA	G	c.1038-8delT	2624	2.20E-01	.	.	.	.	.	Variant in splice region. No effect on the splicing product is predicted by HSF
4	39511530	T	TA	c.664-4dupT	1022	3.19E-01	.	.	.	.	.	Variant in splice region. No effect on the splicing product is predicted by HSF
4	39510283	G	A	c.812-3C>T	6	2.97E-03	.	.	.	.	.	Variant in splice region. No effect on the splicing product is predicted by HSF
4	39511530	TA	T	c.664-4delT	2	4.90E-02	.	.	.	.	.	Variant in splice region. No effect on the splicing product is predicted by HSF
4	39511530	T	TAA	c.664-5_664-4dupTT	1	7.86E-03	.	.	.	.	.	Variant in splice region. No effect on the splicing product is predicted by HSF
4	39510264	A	G	c.828T>C;p.c276c	2689	1.72E-01	.	.	.	.	.	synonymous variant
4	39515736	A	G	c.231T>C;p.D77D	2548	1.69E-01	.	.	.	.	.	synonymous variant
4	39512347	T	C	c.399A>G;p.P133P	963	1.15E-01	.	.	.	.	.	synonymous variant
4	39522995	A	G	c.138T>C;p.N46N	6	2.99E-03	.	.	.	.	.	synonymous variant
4	39523001	C	A	c.132G>T;p.A44A	1	5.69E-04	.	.	.	.	.	synonymous variant

Supplementary Table 3: Homozygous UGDH variants in ExAC: A total of 27 homozygous variants were recorded in ExAC. Twenty of these variants are synonymous, intronic or in the UTR regions. Five variants are within splice site regions but do not affect the splicing products according to the Human Splicing Finder[9]. Only two homozygous missense variants were found, each of them in one single case. Both have low pathogenicity prediction scores than the patients' mutations reported here. No homozygous truncating mutations were present in ExAC.

**Species**	**Gene**	**Forward Primer 5' - 3'**	**Reverse Primer 5' - 3'**	**purpose**
Human	*UGDH*	CTTGCCCAGAGAATAAGCAG	CAAATTCAGAACATCCTTTTGGA	qPCR
Human	*β-ACTIN*	ATGTTTGAGACCTTCACACC	AGGTAGTCAGTCAGGTCCCGGCC	qPCR
Human	*GAPDH*	TGAACCACCAACTGCTTAGC	GGCATGGACTGTGGTCATGAG	qPCR
Human	*PAX6*	CCGTGTGCCTCAACCGTA	CACGGTTTACTGGGTCTGG	qPCR
Human	*TBR2*	AAATGGGTGACCTGTGGCAAAGC	CTCCTGTCTCATCCAGTGGGAA	qPCR
Human	*TUJ1*	TCAGCGTCTACTACAACGAGGC	GCCTGAAGAGATGTCCAAAGGC	qPCR
zebrafish	PCR1	GTATGTGCAGGTGATTGACA	TGTAGGTCACAGGTTTTTGACA	genotyping
zebrafish	PCR2	GACATGAATGAATATCAGAGAAAGAG	AGGAGAAACCCAACAACGC	genotyping
zebrafish	*c-fos*	AACTGTCACGGCGATCTCTT	GCAGGCATGTATGGTTCAGA	qPCR
zebrafish	*gapdh*	GTGGAGTCTACTGGTGTCTT	GTGCAGGAGGCATTGCTTAC	qPCR

Supplementary Table 4: List of primers

Protein	Organism	Dilution factor	Reference
**UGDH**	rabbit	500	Sigma-Aldrich, USA, HPA036657
**GAPDH**	mouse	2000	Santa Cruz Biotechnology Inc., SC 47724
**SOX2**	mouse	200	R&D systems, MAB2018
**TUJ1**	mouse	3000	Biolegend MMS-435P
**GFAP**	rabbit	2500	Dako Z0334
**PCNA**	rabbit	1µg/ml	abcam ab18197

Supplementary Table 5: List of antibodies

Supplementary Note 1

Prevalence estimation of affected UGDH patients based on gnomAD[7].

In a simplified model, the assumption was made that UGDH mutations are spread equally in general population reflecting the worldwide distribution of families in our series. The model ignores the fact that several mutations were found in families with consanguineous background (F1, F5, F16-F25) and therefore likely underestimates the number of affected patients. Based on the 19 detected mutations and the gnomAD[7] spanning 123,136 exomes the allele frequency of identified mutations was assessed. Allele frequencies were available for eight mutations, the other eleven mutations were not represented in gnomAD[7] and are therefore underrepresented in this estimation. Allele frequencies of these eight mutations added up to 1.84×10^{-4} (**Suppl. Table 2**). Further, we assumed that all Loss-of-Function (LoF) variants are pathogenic but hypothesized that biallelic LoF variants are not viable. In gnomAD[7], additional 15 LoF variants were found with a total allele frequency of 8.32×10^{-5} (**Suppl. Table 6**). Additional potentially pathogenic variants were identified by filtering missense variants from gnomAD[7] with the following strict filter criteria:

I) minor allele frequency (MAF) below 1×10^{-4} (rendering the range of already detected pathogenic variants)
II) affecting a residue with similar or higher conservation scores than in the detected mutations (phylop100way>4)
III) consistently pathogenic predictions by SIFT[10], PolyPhen-2 HDIV[11], LRT[12] and MutationTaster[13].

These strict filter criteria resulted in 50 additional potentially pathogenic missense variants with an allele frequency of 4.06×10^{-4} (**Suppl. Table 7**). Based on these results, the allele frequency of UGDH mutations in global population is calculated to be around 6.73×10^{-4}, which would lead to a prevalence of 1:2,210,000 (**Suppl. Table 8**). However, without confirming the potentially pathogenic missense variants identified in gnomAD[7] and just taking into account LoF variants and mutations established in this paper, the allele frequency may be as low as 2.68×10^{-4} (prevalence of 1:14,000,000).

Chr.	Start	End	Ref	Alt	Variant	MAF in GnomAD
4	39501837	39501841	TTCTC	-	exon12 : c.1407_1411del ; p.K469fs	4.09E-06
4	39506054	39506054	C	A	exon10 : c.1246G>T ; p.E416*	4.06E-06
4	39506871	39506871	G	T	exon9 : c.1157C>A ; p.S386*	8.68E-06
4	39506971	39506971	-	A	exon9 : c.1056dupT ; p.I353fs	4.48E-06
4	39507261	39507261	G	-	exon8 : c.1014delC ; p.F338fs	4.11E-06
4	39510254	39510254	C	-	exon7 : c.838delG ; p.D280fs	4.29E-06
4	39511477	39511477	A	-	exon6 : c.714delT ; p.S238fs	4.08E-06
4	39511989	39511989	G	T	exon5 : c.647C>A ; p.S216*	4.06E-06
4	39512105	39512108	TCTG	-	exon5 : c.528_531del ; p.D176fs	4.06E-06
4	39512140	39512140	C	A	exon5 : c.496G>T ; p.G166*	4.06E-06
4	39512316	39512316	-	TCTCT	exon4 : c.429_430insAGAGA ; p.F144fs	4.06E-06
4	39512427	39512427	T	A	exon4 : c.319A>T ; p.K107*	2.44E-05
4	39515800	39515800	C	-	exon3 : c.167delG ; p.G56fs	4.73E-06
4	39523091	39523092	AT	-	exon2 : c.41_42del ; p.Y14fs	4.08E-06
					**Total=**	**8.32E-05**

Supplementary Table 6: Frequency of additional LoF Variants from gnomAD. Abbreviations: Chr = chromosome, Ref = reference, Alt = alternative; n/a: not applicable. All cDNA positions refer to transcript NM_003359. Annotations were obtained using ANNOVAR[6].

Chr	Start	End	Ref	Alt	Variant	MAF in GnomAD	SIFT	PolyPhen-2 HDIV	LRT	Mutation Taster	phyloP 100way [-20;10]
4	39512065	39512065	C	A	exon5 : c.571G>T ; p.A191S	5.28E-05	D	D	D	D	7.266
4	39515773	39515773	C	T	exon3 : c.194G>A ; p.R65Q	3.72E-05	D	D	D	D	6.915
4	39501809	39501809	G	A	exon12 : c.1439C>T ; p.P480L	2.85E-05	D	D	D	D	8.27
4	39512160	39512160	T	C	exon5 : c.476A>G ; p.N159S	2.03E-05	D	D	D	D	7.424
4	39501852	39501852	C	T	exon12 : c.1396G>A ; p.V466M	1.67E-05	D	P	D	D	4.082
4	39505590	39505590	G	A	exon11 : c.1279C>T ; p.R427C	1.22E-05	D	D	D	D	4.851
4	39512097	39512097	A	G	exon5 : c.539T>C ; p.I180T	1.22E-05	D	D	D	D	8.697
4	39515753	39515753	A	G	exon3 : c.214T>C ; p.S72P	9.80E-06	D	D	D	D	8.263
4	39515786	39515786	C	T	exon3 : c.181G>A ; p.V61I	9.34E-06	D	P	D	D	6.915
4	39515720	39515720	C	T	exon3 : c.247G>A ; p.D83N	8.89E-06	D	P	D	D	6.915
4	39506944	39506944	C	T	exon9 : c.1084G>A ; p.A362T	8.63E-06	D	D	D	D	5.507
4	39522985	39522985	G	C	exon2 : c.148C>G ; p.L50V	8.25E-06	D	P	D	D	9.434
4	39511427	39511427	A	G	exon6 : c.764T>C ; p.I255T	8.15E-06	D	D	D	D	8.569
4	39506117	39506117	C	T	exon10 : c.1183G>A ; p.V395M	8.14E-06	D	D	D	D	7.411
4	39505551	39505551	C	T	exon11 : c.1318G>A ; p.D440N	8.12E-06	D	D	D	D	7.741
4	39506072	39506072	C	A	exon10 : c.1228G>T ; p.A410S	8.12E-06	D	D	D	D	7.411
4	39512092	39512092	C	T	exon5 : c.544G>A ; p.G182R	8.12E-06	D	D	D	D	7.266
4	39512348	39512348	G	A	exon4 : c.398C>T ; p.P133L	8.12E-06	D	D	D	D	9.137
4	39515749	39515749	G	C	exon3 : c.218C>G ; p.T73S	4.69E-06	D	P	D	D	6.941
4	39515720	39515720	C	G	exon3 : c.247G>C ; p.D83H	4.44E-06	D	D	D	D	6.915
4	39511385	39511385	C	T	exon6 : c.806G>A ; p.S269N	4.33E-06	D	D	D	D	7.167
4	39523114	39523114	T	A	exon2 : c.19A>T ; p.I7F	4.26E-06	D	D	D	D	5.924
4	39523114	39523114	T	G	exon2 : c.19A>C ; p.I7L	4.26E-06	D	P	D	D	5.924
4	39501776	39501776	T	C	exon12 : c.1472A>G ; p.K491R	4.17E-06	D	P	D	D	6.941
4	39506919	39506919	T	G	exon9 : c.1109A>C ; p.K370T	4.16E-06	D	D	D	D	7.425
4	39507272	39507272	C	T	exon8 : c.1003G>A ; p.G335R	4.11E-06	D	D	D	D	7.487
4	39501786	39501786	G	A	exon12 : c.1462C>T ; p.P488S	4.10E-06	D	D	D	D	8.525
4	39511473	39511473	G	T	exon6 : c.718C>A ; p.L240M	4.08E-06	D	D	D	D	4.278

4	39523074	39523074	C	T	exon2 : c.59G>A ; p.C20Y	4.08E-06	D	D	D	D	7.456
4	39511437	39511437	C	T	exon6 : c.754G>A ; p.A252T	4.07E-06	D	D	D	D	7.167
4	39501810	39501810	G	A	exon12 : c.1438C>T ; p.P480S	4.07E-06	D	P	D	D	8.27
4	39512468	39512468	G	C	exon4 : c.278C>G ; p.T93R	4.07E-06	D	D	D	D	9.137
4	39512477	39512477	T	C	exon4 : c.269A>G ; p.N90S	4.07E-06	D	D	D	D	7.424
4	39512477	39512477	T	G	exon4 : c.269A>C ; p.N90T	4.07E-06	D	D	D	D	7.424
4	39505545	39505545	G	A	exon11 : c.1324C>T ; p.R442W	4.06E-06	D	D	D	D	4.839
4	39505566	39505566	G	C	exon11 : c.1303C>G ; p.P435A	4.06E-06	D	D	D	D	9.768
4	39512004	39512004	G	T	exon5 : c.632C>A ; p.T211N	4.06E-06	D	D	D	D	9.196
4	39512014	39512014	T	A	exon5 : c.622A>T ; p.I208F	4.06E-06	D	D	D	D	7.47
4	39512158	39512158	G	A	exon5 : c.478C>T ; p.P160S	4.06E-06	D	D	D	D	9.137
4	39512414	39512414	G	A	exon4 : c.332C>T ; p.A111V	4.06E-06	D	P	D	D	9.137
4	39512052	39512052	A	G	exon5 : c.584T>C ; p.L195P	4.06E-06	D	D	D	D	8.697
4	39512109	39512109	T	C	exon5 : c.527A>G ; p.D176G	4.06E-06	D	D	D	D	7.47
4	39512113	39512113	G	T	exon5 : c.523C>A ; p.P175T	4.06E-06	D	D	D	D	9.196
4	39512136	39512136	G	A	exon5 : c.500C>T ; p.T167I	4.06E-06	D	D	D	D	9.137
4	39512145	39512145	G	T	exon5 : c.491C>A ; p.A164E	4.06E-06	D	D	D	D	9.137
4	39512345	39512345	A	C	exon4 : c.401T>G ; p.V134G	4.06E-06	D	D	D	D	8.642
4	39512345	39512345	A	G	exon4 : c.401T>C ; p.V134A	4.06E-06	D	P	D	D	8.642
4	39512351	39512351	A	C	exon4 : c.395T>G ; p.V132G	4.06E-06	D	D	D	D	8.642
4	39512367	39512367	T	G	exon4 : c.379A>C ; p.T127P	4.06E-06	D	P	D	D	5.693
4	39512376	39512376	T	C	exon4 : c.370A>G ; p.K124E	4.06E-06	D	D	D	D	7.424
					Total =	**4.06E-04**					

Supplementary Table 7: Additional potentially pathogenic missense variants from gnomAD. Abbreviations: Chr = chromosome, Ref = reference, Alt = alternative; n/a: not applicable; D = damaging (SIFT) / probably damaging (Polyphen2) / deleterious (LRT) / disease causing (Mutation Taster); P = possibly damaging. All cDNA positions refer to transcript NM_003359. Annotations were obtained using ANNOVAR[6].

	total MAF in gnomAD	estimated prevalence	1 patient per
identified mutations	1.84E-04		
additional LoF	8.32E-05		
additional potentially pathogenic missense variants	4.06E-04		
total	6.73E-04	4.53E-07	2.21E+06
total without potentially pathogenic missense variants	2.68E-04	7.16E-08	1.40E+07

Supplementary Table 8: Prevalence estimation based on gnomAD. Abbreviation**:** MAF: minor allele frequency.

Supplementary References

1. Goujon, M. *et al.* A new bioinformatics analysis tools framework at EMBL-EBI. *Nucleic Acids Res.* **38**, 695–699 (2010).
2. Sievers, F. *et al.* Fast, scalable generation of high-quality protein multiple sequence alignments using Clustal Omega. *Mol. Syst. Biol.* **7**, 539–539 (2014).
3. Sommer, B. J., Barycki, J. J. & Simpson, M. A. Characterization of human UDP-glucose dehydrogenase: CYS-276 is required for the second of two successive oxidations. *J. Biol. Chem.* **279**, 23590–23596 (2004).
4. Egger, S., Chaikuad, A., Kavanagh, K. L., Oppermann, U. & Nidetzky, B. Structure and mechanism of human UDP-glucose 6-dehydrogenase. *J. Biol. Chem.* **286**, 23877–23887 (2011).
5. Guex, N. & Peitsch, M. C. SWISS-MODEL and the Swiss-PdbViewer: An environment for comparative protein modeling. *Electrophoresis* **18**, 2714–2723 (1997).
6. Wang, K., Li, M. & Hakonarson, H. ANNOVAR: Functional annotation of genetic variants from high-throughput sequencing data. *Nucleic Acids Res.* **38**, 1–7 (2010).
7. Lek, M. *et al.* Analysis of protein-coding genetic variation in 60,706 humans. *Nature* **536**, 285–291 (2016).
8. Pires, D. E. V, Ascher, D. B. & Blundell, T. L. DUET: A server for predicting effects of mutations on protein stability using an integrated computational approach. *Nucleic Acids Res.* **42**, 314–319 (2014).
9. Desmet, F. O. *et al.* Human Splicing Finder: An online bioinformatics tool to predict splicing signals. *Nucleic Acids Res.* **37**, 1–14 (2009).
10. Kumar, P., Henikoff, S. & Ng, P. C. Predicting the effects of coding non-synonymous variants on protein function using the SIFT algorithm. *Nat. Protoc.* **4**, 1073–1081 (2009).
11. Adzhubei, I. A. *et al.* A method and server for predicting damaging missense mutations. *Nat. Methods* **7**, 248–249 (2010).
12. Chun, S. & Fay, J. C. Identification of deleterious mutations within three human genomes. *Identif. deleterious Mutat. within three Hum. genomes.* **19**, 1553–1561 (2009).
13. Schwarz, J. M., Rödelsperger, C., Schuelke, M. & Seelow, D. MutationTaster evaluates disease-causing potential of sequence alterations. *Nat. Methods* **7**, 575–576 (2010).
