# Supplementary material for: Loss-of-function mutations in UDP-Glucose 6-Dehydrogenase cause recessive developmental epileptic encephalopathy
Source: Nat Commun. 2020 Jan 30;11:595. doi: 10.1038/s41467-020-14360-7 (PMC6992768; doi:10.1038/s41467-020-14360-7)
Supplement: Supplementary file 3 — Description of Additional Supplementary Files [file 41467_2020_14360_MOESM3_ESM.pdf]

Title: Supplementary Data 1: Clinical findings and course of disease in patients with UGDH mutations.

Description: Abbreviations: hom: homozygous; M: male; F: Female; n/a: not applicable; ND: non determined; ID: intellectual disability; g-tube: gastrostomy tube; NJ: nasojejunal. ^: DNA from father not available; †: patient deceased. * multiple congenital anomalies in F16-II:1: prenatal polyhydramnios; multiple ocular anomalies (bilateral cataracts, multiple bilateral lens colobomas, bilateral microphthalmia, hypoplastic iris, iris and lenticular vascularization, bilateral anterior segment dysgenesis, posterior synechiae bilaterally secondary to neovascularization); megacystis; neurogenic bladder; moderate hiatal hernia; camptodactyly of 3rd and 4th fingers and overriding 2nd and 4th digits on the right hand; long tapered fingers; skeletal survey showed overlapping of the parietal bones and mild elongation of the 2nd through 5th fingers.
