# Supplementary figures and images for: Loss-of-function mutations in UDP-Glucose 6-Dehydrogenase cause recessive developmental epileptic encephalopathy
Source: Nat Commun. 2020 Jan 30;11:595. doi: 10.1038/s41467-020-14360-7 (PMC6992768; doi:10.1038/s41467-020-14360-7)

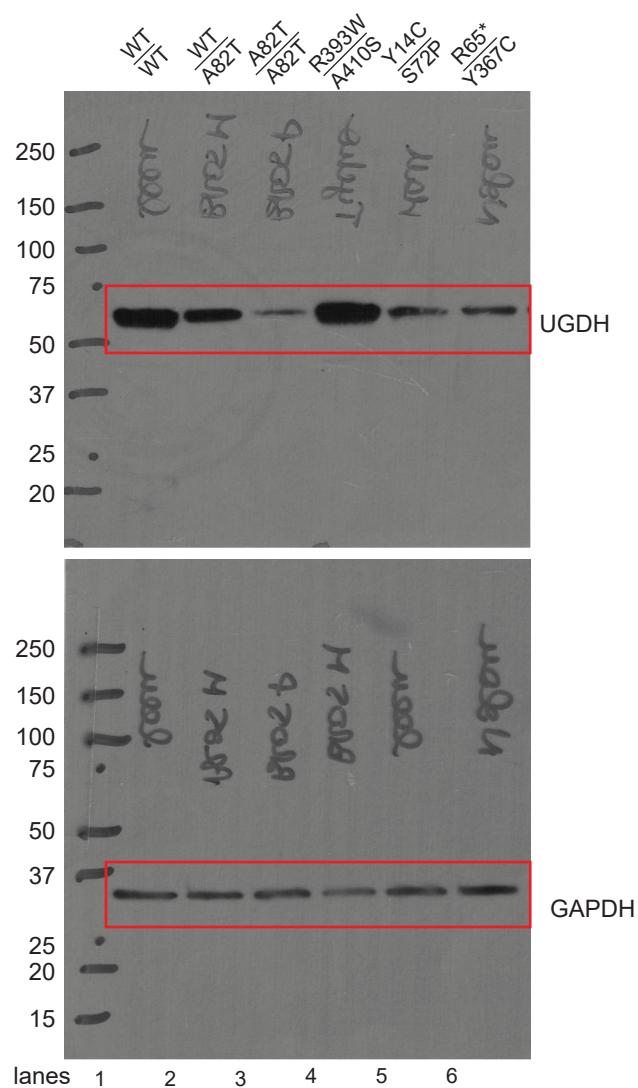

**Figure 2c**

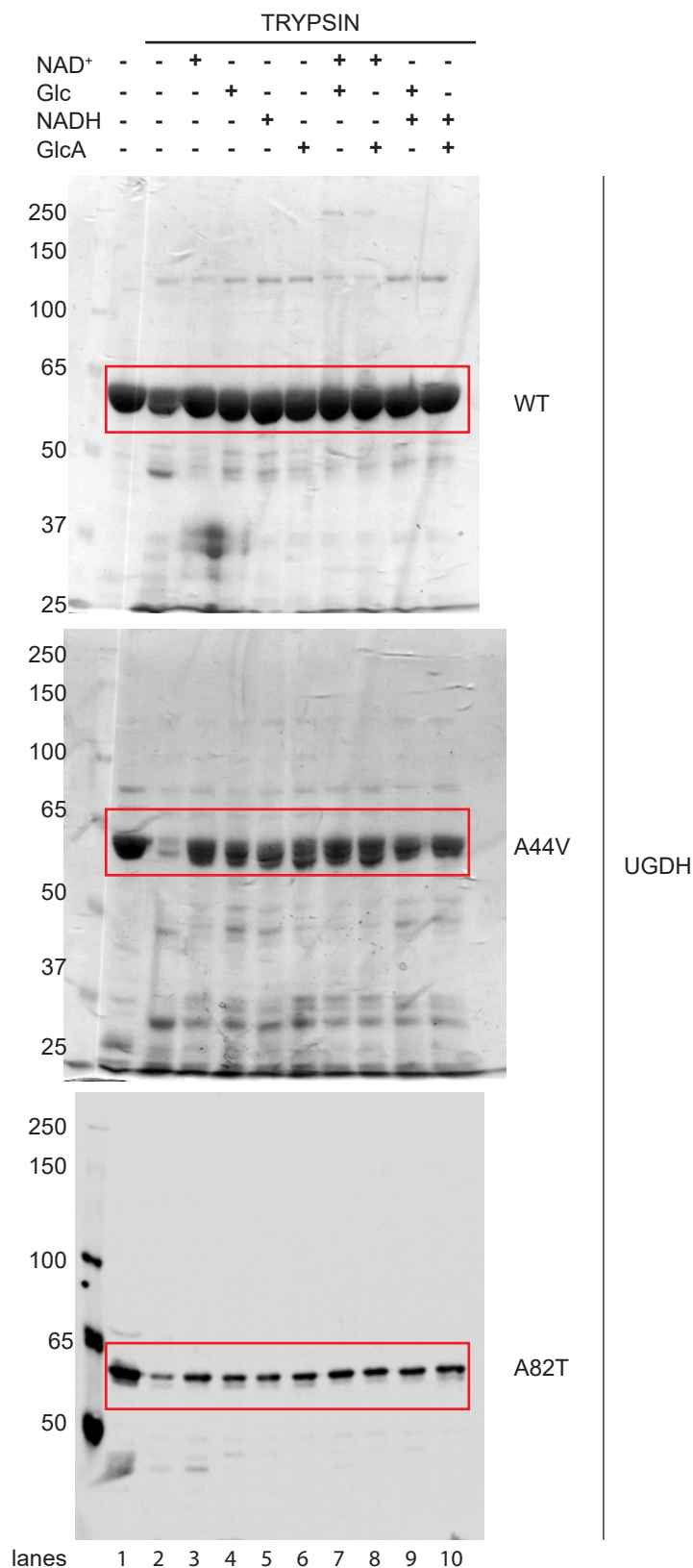

**Figure 3a**

Supplement: Supplementary file 4 — Source Data [file 41467_2020_14360_MOESM4_ESM.zip › Hengel&Bosso-Lefevre_Schols&Reversade_2020_Source Data file 1.pdf]
